# Targeted repression of *topA* by CRISPRi reveals a critical function for balanced DNA topoisomerase I activity in the *Chlamydia trachomatis* developmental cycle

**DOI:** 10.1128/mbio.02584-23

**Published:** 2024-01-24

**Authors:** Li Shen, Leiqiong Gao, Abigail R. Swoboda, Scot P. Ouellette

**Affiliations:** 1 Department of Microbiology, Immunology and Parasitology, Louisiana State University Health Sciences Center, New Orleans, Louisiana, USA; 2 Department of Pathology, Microbiology, and Immunology, University of Nebraska Medical Center, Omaha, Nebraska, USA; Yale University School of Medicine, New Haven, Connecticut, USA; National Institute of Allergy and Infectious Diseases, Hamilton, Montana, USA

**Keywords:** *Chlamydia trachomatis*, DNA topoisomerase, *topA*, CRISPRi, dCas12, bacterial developmental cycle, gene expression, antibacterial mechanism, fluoroquinolone, moxifloxacin

## Abstract

*Chlamydia trachomatis* is an obligate intracellular bacterium that is responsible for the most prevalent bacterial sexually transmitted infection. Changes in DNA topology in this pathogen have been linked to its pathogenicity-associated developmental cycle. Here, evidence is provided that the balanced activity of DNA topoisomerases contributes to controlling *Chlamydia* developmental processes. Utilizing catalytically inactivated Cas12 (dCas12)-based clustered regularly interspaced short palindromic repeats interference (CRISPRi) technology, we demonstrate targeted knockdown of chromosomal *topA* transcription in *C. trachomatis* without detected toxicity of dCas12. Repression of *topA* impaired the developmental cycle of *C. trachomatis* mostly through disruption of its differentiation from a replicative form to an infectious form. Consistent with this, expression of late developmental genes of *C. trachomatis* was downregulated, while early genes maintained their expression. Importantly, the developmental defect associated with *topA* knockdown was rescued by overexpressing *topA* at an appropriate degree and time, directly linking the growth patterns to the levels of *topA* expression. Interestingly, *topA* knockdown had effects on DNA gyrase expression, indicating a potential compensatory mechanism for survival to offset TopA deficiency. *C. trachomatis* with *topA* knocked down displayed hypersensitivity to moxifloxacin that targets DNA gyrase in comparison with the wild type. These data underscore the requirement of integrated topoisomerase actions to support the essential developmental and transcriptional processes of *C. trachomatis*.

**IMPORTANCE:**

We used genetic and chemical tools to demonstrate the relationship of topoisomerase activities and their obligatory role for the chlamydial developmental cycle. Successfully targeting the essential gene *topA* with a CRISPRi approach, using dCas12, in *C. trachomatis* indicates that this method will facilitate the characterization of the essential genome. These findings have an important impact on our understanding of the mechanisms by which well-balanced topoisomerase functions in adaptation of *C. trachomatis* to unfavorable growth conditions imposed by antibiotics.

## INTRODUCTION

A group of enzymes, namely, DNA topoisomerases (Topos), act to correct the altered DNA topology that occurs during DNA replication, transcription, and recombination by causing temporary breaks on the DNA helix to prevent excessive supercoiling that is deleterious (1, 2). Most pathogenic bacteria encode two classes of Topos: (i) type IA (e.g.,TopoI or TopA), which cleaves and rejoins single-strand DNA independently of ATP, and (ii) type II (e.g., DNA gyrase and TopoIV), which exerts its effects through ATP-dependent double-strand cleavage. An accepted model in *Escherichia coli* is that the concerted action of these Topos dictate the topological properties of DNA (3–5). Whereas gyrase holoenzyme (composed of two GyrA and two GyrB subunits) negatively supercoils, monomeric TopA removes excessive negative supercoils and works along with gyrase to control the superhelical density of the chromosome. The TopoIV holoenzyme (composed of two ParC and two ParE subunits) disentangles replicated DNA and enables segregation of daughter chromosomes. Because Topos are ubiquitous, they are considered to be essential for bacterial viability. Fluoroquinolone antibiotics, like moxifloxacin (Mox), target gyrase and TopoIV in many bacteria and are widely prescribed to treat serious infections associated with *Enterobacterales*, *Mycobacterium tuberculosis*, *Pseudomonas aeruginosa*, *S. pneumoniae*, *Moraxella catarrhalis*, *Chlamydia*, *Mycoplasma*, and *Staphylococcus* species (6–8). However, emerging mutations in genes encoding gyrase or TopoIV have conferred moxifloxacin resistance during the last decades, presenting an urgent need for discovery of new classes of antibacterial compounds (9, 10). Characterizing the function of Topos in, and their effects on, bacterial physiology may facilitate the development of new antibacterial therapies.

*Chlamydia trachomatis* is a Gram-negative bacterial parasite that is the leading cause of bacterial sexually transmitted infections worldwide (11). *C. trachomatis* primarily infects human mucosal epithelial cells, where it grows in a membrane-bound vacuole (named as an inclusion) and exists as functionally and structurally distinct forms (12, 13). These forms mainly include (i) the noninfectious, replicative reticulate body (RB) that has a dispersed chromatin structure, and (ii) the infectious, non-replicative elementary body (EB) that is typified by DNA condensation. EB differentiation to RB is detected by 2 h post infection (h pi), and this is followed by rapid RB multiplication via an asymmetric polarized division mechanism starting at approximately 10 h pi (14–16). RBs begin to asynchronously undergo secondary differentiation into EBs starting at ~16 h pi, depending on the serovar. However, when stressed, *Chlamydia* can enter an aberrant growth mode called persistence *in vitro*. Signals that trigger the variations of *C. trachomatis* development remain unknown, but one striking change is the DNA supercoiling (13, 17, 18). This can be exemplified by the superhelicity of the plasmid that peaks at ~24 h pi and is much higher than that at the early or late developmental stages of *C. trachomatis*. These findings raise the question of how DNA topology is regulated and what the consequences of topological changes are for the chlamydial developmental cycle.

*C. trachomatis* possesses a small chromosome of ~1.0 Mbp and a plasmid of 7.5 kbp (19). It has been proposed that chlamydial DNA topology is managed by three Topos (gyrase, TopoIV, and TopA) and certain DNA binding proteins like HctB, a histone-like protein (13, 17, 18, 20). Located in three separate operons on the chlamydial chromosome, the Topo encoded genes are transcribed by RNA polymerase (RNAP) containing the major sigma factor σ^66^ (19, 21) and are expressed in temporal fashion through a not-yet-identified mechanism. *In vitro*, individual recombinant Topo enzymes modified the superhelical density of plasmid DNA and affected transcription from selected promoters using the plasmid DNA as templates (18, 20, 21). *C. trachomatis* was sensitive to aminocoumarin (i.e., novobiocin), which targets GyrB, altering transcription of select genes as analyzed by reverse transcription quantitative PCR (RT-qPCR) (21). These studies indirectly indicate that developmentally regulated changes in DNA topology and Topo expression occur in *Chlamydia*. However, they did not adequately address the question regarding how TopA, in conjunction with type II Topos, directly influences the chlamydial developmental cycle. The lack, until recently, of genetic tools is the main cause of such knowledge gaps.

The purposes of the current study were to (i) determine the role of TopA in the chlamydial developmental cycle *in vivo* using clustered regularly interspaced short palindromic repeats interference (CRISPRi) for targeted knockdown of *topA*, and (ii) investigate the effects of DNA relaxation on chlamydial developmental cycle by overexpressing TopA or using moxifloxacin, a widely used pharmacological gyrase/TopoIV inhibitor. This design allows for investigation of how retention of or interference with TopA function alone or in combination with inhibition of gyrase and TopoIV affects key developmental events in *C. trachomatis*. Through developmental pattern and morphology measurements, this work describes the first detailed phenotype of TopA deficiency, or interference, in *Chlamydia* and indicates the importance of carefully balanced Topo activities for the chlamydial adaptive response to changing levels of DNA supercoiling. It further establishes the utility of CRISPRi in understanding essential gene function in this important pathogen.

## RESULTS

### Chromosomal *topA* can be targeted using CRISPRi

**Fig 1:**
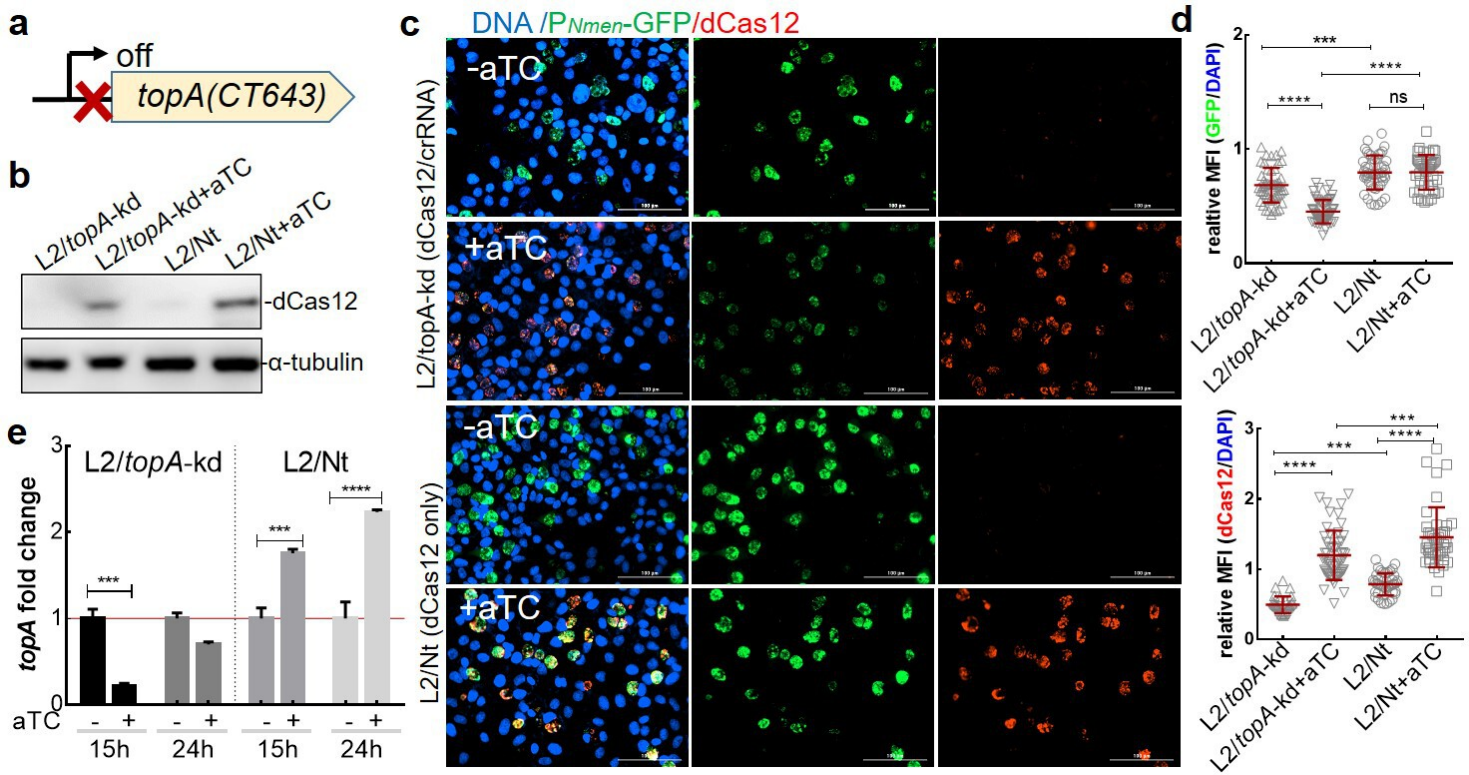
Conditional repression of *topA* transcription in *C. trachomatis*. (a) Schematic representation of the strategy used to make a targeted *topA* knockdown through dCas12 and a specific crRNA, whose targeting site is indicated by the red X. (b) Immunoblot for dCas12 in L2/*topA*-kd or L2/Nt-infected HeLa cells in the absence or presence of aTC (10 ng/mL) added at 0 h pi. Cells were sampled at 24 h pi for immunoblotting with rabbit anti-dCas12 primary antibody and anti-rabbit horseradish peroxidase-conjugated secondary antibody. Host cell α-tubulin was probed with a mouse anti-tubulin antibody and used as a protein loading control. (c) Immunofluorescence micrograph of *C. trachomatis* showing the relatively low levels of GFP in L2/*topA-*kd compared to L2/Nt. *C. trachomatis*-infected HeLa cells were grown in the absence or presence of aTC (10 ng/mL), fixed at 40 h pi, and subjected to IFA with rabbit anti-dCas12 antibody and then Alexa Fluor 568-conjugated goat anti-rabbit IgG to visualize. Host cell and bacterial DNA were counterstained with DAPI. The automated images were obtained using Cytation 1 and analyzed by the software Gen-5. *C. trachomatis* expressing GFP (green), dCas12 (red), and DAPI-stained DNA (blue) is shown. (d) The quantitative data from panel c are presented as relative MFI of GFP (upper panel) and dCas12 (lower panel); each is normalized to DAPI in the same inclusion. The number of the inclusion counts per condition is 43 ± 9. Scale bar = 100 µm. Statistical significance was determined by one-way ANOVA followed by Tukey’s post hoc test. ***P ≤0.001, ****P ≤ 0.0001. (e) Fold change in *topA* transcript levels. RT-qPCR was performed with *C. trachomatis*-infected cells grown under inducing (+aTC) or mock inducing (−aTC) conditions starting from 4 h pi for 11 h (to 15 h pi) and 20 h (to 24 h pi). Quantified *topA*-specific transcripts were normalized to the gDNA value from respective cultures using primers targeting *topA*. The data are presented as the ratio of relative *topA* transcript in the presence of aTC to that in the absence of aTC, which is set at 1 as shown by a red line. Data from three biological replicates of an experiment are shown. At least three independent experiments were performed. Statistical significance was determined by two-way ANOVA followed by Tukey’s post hoc test. ***P ≤ 0.001, ****P ≤ 0.0001. ANOVA, analysis of variance; DAPI, 4′,6-diamidino-2-phenylindole; IFA, immunofluorescence assay; MFI, mean florescence intensity; ns, no significance.

Recently, CRISPRi has been used for targeted gene inhibition in *C. trachomatis* (22, 23). To elucidate the role of TopA, we investigated whether it was possible to target chromosomal *topA* using CRISPRi. We created a new spectinomycin-resistance encoding vector, pBOMBL12CRia(*topA*)::L2 (Fig. S1; Table S1), that used a modified pBOMB4-Tet-mCherry backbone (24). The pBOMBL12CRia(*topA*)::L2 contains (i) a tetracycline promoter (P*_tet_*)/repressor controlled catalytically inactivated Cas12 (*dCas12*) gene with a weakened ribosome binding site, (ii) a specific *topA*-targeting crRNA sequence controlled by a weakened, constitutive chlamydial *dnaK* promoter, (iii) a *Neisseria meningitis* promoter (P*_Nmen_*)‐linked *gfp* gene (25), and (iv) a spectinomycin/streptomycin resistance gene, *aadA*, to facilitate selection for *C. trachomatis* transformants. The design permits that a specific crRNA directs anhydrotetracycline (aTC)-inducible dCas12 to a specific DNA target (here, the promoter region of *topA* on the *C. trachomatis* chromosome), where it represses transcription (Fig. 1a). The control vector, designated as pBOMBL12CRia(NT)::L2, contained the same components except for the *topA*-specific crRNA, which was replaced with a scrambled sequence with no homology to any chlamydial sequence. Each vector was transformed into *C. trachomatis* serovar L2 -pL2 (22, 26), resulting in strains L2/*topA*‐kd and L2/Nt; both were then used individually to infect HeLa cells.

We first confirmed that dCas12 expression was induced by addition of aTC. *C. trachomatis*-infected HeLa cells cultured in medium without or with aTC (at 10 ng/mL or ~5 nM) immediately after infection. The small amount of aTC used did not have a significant inhibitory effect on the growth of *C. trachomatis* (22, 24, 27). Expression of dCas12 was examined by indirect immunofluorescence assay (IFA) in single cells and by immunoblotting analysis with the lysates of the cell population. We detected the induction of dCas12 expression after adding aTC in L2/*topA*‐kd or L2/Nt cultures (Fig. 1b through d). However, there was a much lower GFP signal in L2/*topA*‐kd inclusions than in that of L2/Nt inclusions. These results indicate that dCas12 is inducible and stably present, causing different phenotypes in *C. trachomatis* L2/*topA*‐kd and L2/Nt.

We next determined whether targeted *topA* knockdown occurred in L2/*topA*-kd using RT-qPCR. Because native *topA* is expressed preferentially at the mid-stage, with transcript levels peaking at 14–16 h pi as described previously (21, 28), we expected that aTC addition at 4 h pi would have maximal efficiency in *topA* repression induced by CRISPRi. Indeed, the addition of aTC decreased *topA* transcripts by ~80% and ~30% at 15 and 24 h pi, respectively, in *C. trachomatis* L2/*topA*-kd (Fig. 1e). In contrast, *topA* transcripts were increased approximately twofold in strain L2/Nt at 24 h pi after adding aTC, suggesting that the dCas12 induction alone did not impair *topA* transcription.

These data demonstrate that *topA* transcription in *C. trachomatis* can be conditionally repressed using CRISPRi. Importantly, both *topA*-specific crRNA and the inducible dCas12 expression are necessary and sufficient for successful *topA* knockdown. Repression of *topA* is likely not due to an off-target effect, as we used a crRNA sequence that is unique to chlamydial *topA* as determined by analysis of the entire sequence of *C. trachomatis*. The maximum homology of the crRNA to either the chromosome or plasmid was 14 of 21 bp with no PAM sequence that would target the correct strand in a site likely to result in transcriptional repression. In addition, there was no decrease in *topA* transcripts in the control strain, L2/Nt, and *topA* interference only occurred in strain L2/*topA*-kd in the presence of aTC.

### TopA activity is critical for the *C. trachomatis* developmental cycle

**Fig 2:**
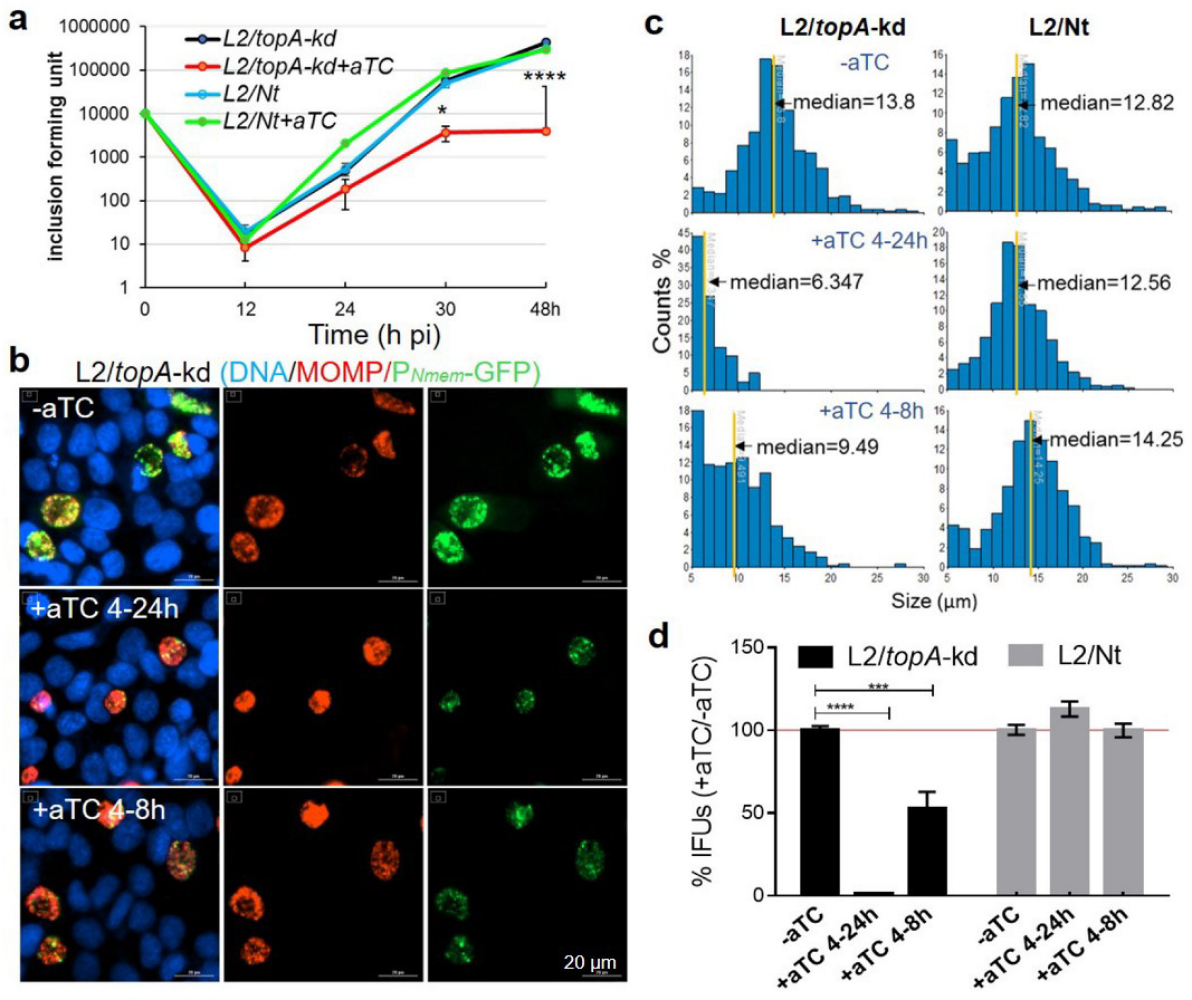
Targeted knockdown of *topA* decelerates the developmental cycle of *C. trachomatis*. (a) One-step growth curve of *C. trachomatis*. HeLa cells were infected with *C. trachomatis* L2/*topA*-kd or L2/Nt at the dose that resulted in 40% cell infection (multiplicity of infection = 0.4) and cultured in the absence or presence of aTC (10 ng/mL) added at 0 h pi. Cells sampled at 0, 12, 24, 30, or 48 h pi (time h pi, *x*-axis) were used for determination of IFUs (*y*-axis) on fresh HeLa monolayers. IFU values are expressed as the mean ± SD from triplicate samples. Experiment was repeated at least three times. (b) Representative immunofluorescence images of *C. trachomatis* L2/*topA*-kd. Infected HeLa cells were grown under the conditions of dCas12 induction for 20 h (+aTC4-24 h), transient induction from 4 to 8 h pi (+aTC 4 h-8h), or mock induction (−aTC). Fixed cells at 24 h pi were immunolabeled with mouse monoclonal antibody to *C. trachomatis* L2 MOMP and visualized with Alexa fluor 568-conjugated goat anti-mouse IgG. The automated images were acquired and analyzed using cytation1 and Gen5. The DAPI-stained DNA (blue) and *C. trachomatis* expressing GFP (green) and MOMP (red) are shown. Scale bar = 20 µm. (c) Histogram displays the distribution of individual *C. trachomatis* inclusion sizes *y*-axis, percentage of inclusion counts; *x*-axis, inclusion size [μm]). Graph shows inclusion measurement of one representative well with nine different fields per condition. The yellow lines indicate median inclusion size counted. Three independent trials were performed with similar results. (d) Relative IFUs in the absence or presence of aTC for 20 or 4 h. Triplicate results in a representative experiment are shown as mean ± SD. Values are presented as the ratio of IFU from dCas12 induced sample to that from respective mock induction sample, which is set at 1. At least four independent experiments were performed (also see Fig. S2, in which actual IFU values were presented). Statistical significance in all panels was determined by two-way ANOVA followed by Tukey’s post hoc test. *P ≤ 0.05*,* ***P ≤ 0.001, ****P ≤ 0.0001. IFU, inclusion-forming unit; MOMP, major outer membrane protein.

We noted that, under dCas12-inducing conditions, the levels of dCas12 expression and the GFP signal in strain L2/*topA*-kd were qualitatively lower than those in L2/Nt (Fig. 1). This could have two non-mutually exclusive interpretations. First, CRISPRi-induced *topA* repression interferes with *Chlamydia* growth and, second, reduced TopA expression may interfere with P*_Nmen_*-GFP expression from the plasmid. To test these possibilities, we first generated a series of growth curves to examine chlamydial developmental kinetics in the absence or presence of aTC. Growth curves were created by enumeration of the inclusion-forming unit (IFU) (equivalent to infectious EB yield) at different time points along the 48-h experimental period after passaging onto a fresh cell monolayer. In the absence of aTC, there was no significant difference in the EB yields between the L2/*topA*-kd and L2/Nt at 24 h pi and thereafter (Fig. 2a). Adding aTC (10 ng/mL) to L2/*topA*-kd culture resulted in ~1/2 log decrease in EB yields at 24 h pi and ~l log less at 30 h pi than those from cultures lacking aTC (*P* < 0.05). The EB amounts remained ~2 log lower at 48 h pi (*P* < 0.0001), indicating an incomplete chlamydial developmental cycle or the formation of non-infectious forms in L2/*topA*-kd culture. Despite the plentiful dCas12 induction in L2/Nt, EBs accumulated to levels similar to that of the uninduced conditions (*P* > 0.05). Thus, dCas12 induction alone has a minimal impact on chlamydial growth. Rather, it is the combination of inducible dCas12 and the targeted crRNA to mediate *topA* repression that causes the growth defect of *C. trachomatis*.

*C. trachomatis* lives within the intracellular inclusion niche, whose expansion mirrors the pathogen-host interactions. We sought to examine whether the duration of *topA* knockdown affected the inclusion morphology. Two different culture conditions were chosen: dCas12 induction for 20 h (from 4 to 24 h pi to cover the early and mid-stages) or only 4 h (from 4 to 8 h pi to cover the early stage prior to EB accumulation). The size of chlamydial inclusions was measured following IFA with antibody to the major outer membrane protein (MOMP); in parallel, progeny EB yield was assessed. When dCas12 was induced for 20 h (+aTC 4–24 h), L2/*topA*-kd formed smaller inclusions, unlike the control L2/Nt that displayed “normal” large inclusions (Fig. 2b and c). Consistent with the smaller inclusion size, progeny EBs were decreased by ~85% in L2/*topA*-kd culture (Fig. 2d and Fig. S2). Induction of dCas12 for 4 h (+aTC 4–8 h) had little effect on the inclusion sizes but decreased EB yield by ~40%, indicating that even a short window of *topA* knockdown can have a measurable effect on chlamydial development. None of these changes were observed for strain L2/Nt. These data suggest that *C. trachomatis* is sensitive to either transient or prolonged *topA* repression induced by CRISPRi, highlighting the critical role of TopA in supporting the intracellular developmental cycle of *C. trachomatis*.

### P*_Nmen_*-GFP expression is reduced upon *topA* knockdown

**Fig 3:**
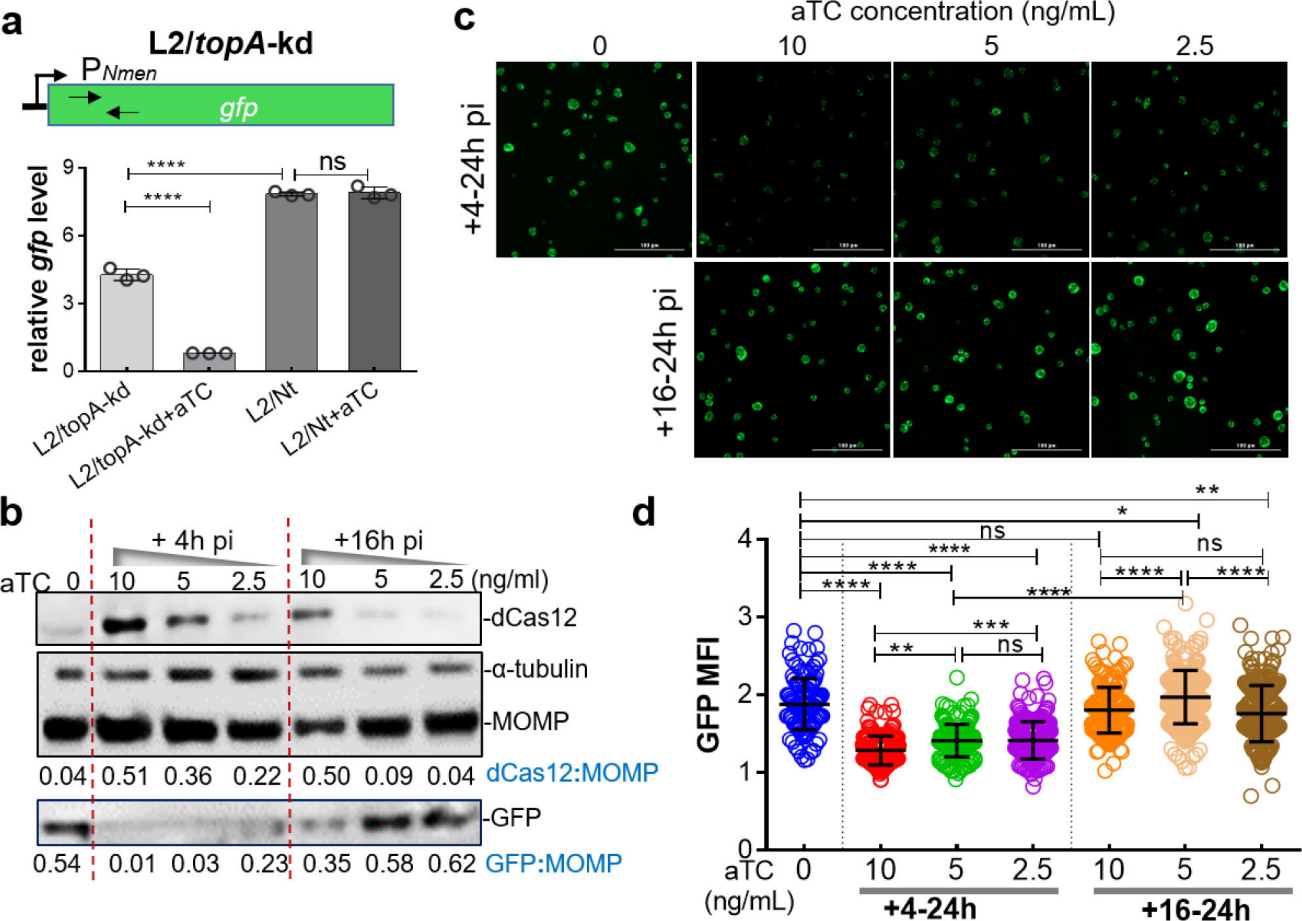
Dose- and time-dependent effects of targeted *topA* knockdown on P*_Nmen_-gfp* expression in *C. trachomatis* L2/*topA*-kd. (a) Quantification of *gfp* expression using RT-qPCR. The sites of primers used to detect *gfp* from the sample cDNA are indicated (also see Table S2). The *gfp* mRNA concentrations were normalized to the gDNA levels as determined by qPCR targeting *tufA* and presented as mean ± SD of three biological replicates. (b) Immunoblotting analysis of dCas12, MOMP, and GFP expression with infected cells grown at increasing concentration of aTC added at 4 h pi. Densitometry of the blot was assessed using ImageJ. Values are presented as the density of the dCas12 band or GFP band normalized to the MOMP band from the same sample. Host cell α-tubulin was used as protein loading control. Note: a small amount of dCas12 leaky expression was detected in the absence of aTC. (c) Live-cell images of *C. trachomatis*. HeLa cells were infected with *C. trachomatis* L2/*topA*-kd at a multiplicity of infection of ~0.4 and cultured in an aTC-free medium. Increasing concentrations of aTC (0, 2.5, 5, or 10 ng/mL) were added starting at 4 or 16 h pi. Automated imaging acquisition was performed at 24 h pi under the same exposure conditions with Cytation 1. Scale bar = 100 µm. (d) Measurement of GFP MFI in *C. trachomatis*-infected cells grown in the absence or presence of aTC. Individual inclusions were analyzed using Gen5 software. The values are presented as mean ± SD from the inclusion numbers equal to 213 ± 41 per condition in replicate wells. Statistical significance in all panels was determined by one-way ANOVA followed by Tukey’s post hoc test. *P ≤ 0.05, ** P ≤ 0.01, ***P  ≤  0.001, ****P  ≤  0.0001. ns, no significance.

As noted above, we observed a weakened P*_Nmen_*-driven GFP signal in *C. trachomatis* L2/*topA*-kd upon *topA* knockdown. Using RT-qPCR, we measured a decrease in *gfp* transcripts in L2/*topA*-kd, but not in L2/Nt, under dCas12-inducing conditions (Fig. 3a). Thus, changes in P*_Nmen_-gfp* levels represent another measurable impact mediated by CRISPRi-induced *topA* repression.

We next determined to what degree repression of *topA*, measured as a function of dCas12 expression, altered P*_Nmen_-gfp* expression in *C. trachomatis*. This is worthwhile because robust P*_Nmen_-gfp* expression could serve as a tool to facilitate monitoring chlamydial development (29, 30). *C. trachomatis* L2/*topA*-kd-infected cells grown in the presence of increasing concentrations of aTC were used to assess the GFP mean florescence intensity (MFI) using quantitative microscopy. This assay is based on automated live-cell imaging in combination with green fluorescence and bright light detection. The intensity ratio of GFP to bright light field in an individual inclusion was calculated to estimate the GFP MFI. Immunoblotting analysis of respective cell lysates was performed to quantify the protein levels of dCas12 and GFP along with that of the MOMP for normalization.

When aTC (at the concentrations from 0 to 10 ng/mL) was added to L2/*topA*-kd culture starting from 4 h pi, we observed effects of *topA* repression at 24 h pi: the larger aTC dose used, the more dCas12 produced, and the lower GFP signal or protein product detected (Fig. 3b through d). Lesser effects on dCas12 induction and GFP decrease were detected when aTC was added starting at 16 h pi. The decreased GFP in L2/*topA*-kd was consistent with the reduced EB yields (Fig. 2). Neither GFP levels nor EB yields were decreased in the control strain, L2/Nt (Fig. S3; Fig. 2). These data indicate that the aTC dose, as well as the time of addition and its duration used, affects the expression level of dCas12 that is directly linked to the degree of *topA* repression, and subsequently the GFP levels, in L2/*topA*-kd.

Because RBs begin to asynchronously differentiate to EBs at ~16 h pi and extant RB and EB forms co-exist at later time points, the differences in the time-related efficiency of dCas12 induction suggest that the growth or physiological state of *C. trachomatis* plays a role in determination of dCas12 induction. For example, it is unlikely that EBs express dCas12 to any great degree, given their reduced metabolic activity and macromolecule synthesis (31, 32). Alternatively, the chlamydial developmental forms may have different responses to CRISPRi-induced *topA* repression, as analyzed by measuring P*_Nmen_*-GFP levels. Thus, in addition to the effects related to EB yield and inclusion expansion, changes in P*_Nmen_*-GFP levels allow for sensitive detection of *topA* repression-mediated inhibition of *C. trachomatis* development.

### Targeted *topA* knockdown interrupts RB-to-EB differentiation

**Fig 4:**
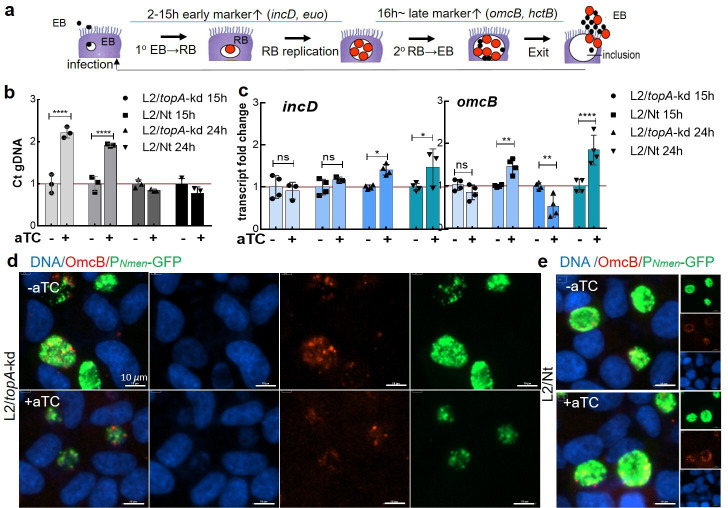
Secondary differentiation of RB to EB is impaired by *topA* knockdown in L2/*topA*-kd. (a) Schematic representation of the chlamydial developmental cycle and its coupled expression of RB- or EB-related markers. (b) Analysis of *C. trachomatis* gDNA in the absence or presence of aTC using real-time qPCR targeting the *tufA* gene. Values are presented as the ratio of chlamydial gDNA copy numbers per nanogram DNA in +aTC sample to that of −aTC sample, which is set at 1. Triplicate results in a representative experiment are shown. At least two independent experiments were performed. (c) Quantification of transcripts of *omcB* or *incD* in *C. trachomatis* using RT-qPCR. The levels of transcripts were normalized to the gDNA levels as determined by qPCR with the same primer pair. The values are presented as mean ± SD of four biological replicates. (**d and **e) Immunofluorescent micrographs of *C. trachomatis* expressing OmcB. HeLa cells were infected with *C. trachomatis* L2/*topA*-kd (d) or L2/Nt (e), cultured in the absence (−aTC) or presence of aTC (+aTC, 10 ng/mL) for 20 h pi starting at 4 h pi. After 24 h pi, infected cells were fixed, processed, and used for IFA by immunolabeling with rabbit polyclonal antibody to *C. trachomatis* OmcB and visualized with Alexa fluor 568-conjugated goat anti-rabbit antibody. DAPI*-*counterstained DNA (blue) and *C. trachomatis* expressing GFP (green) and OmcB (red) are shown. Scale bar = 10 µm. Quantitative analysis of OmcB signal has been omitted as OmcB is a secretable protein that can be associated or not with the inclusion. Statistical significance was determined by two-way ANOVA followed by Tukey’s post hoc test. *P ≤ 0.05, **P ≤ 0.01, ****P ≤ 0.0001. ns, no significance.

It is important to determine what process of chlamydial development is impaired by *topA* repression. A chlamydial developmental cycle typically involves the successive events of EB-to-RB differentiation at early stage, RB multiplication at mid-cycle, and RB-to-EB differentiation at late stage (Fig. 4a). Because the IFU assay is unable to assess non-infectious forms, such as RBs, we performed qPCR to quantify total *C. trachomatis* gDNA, which is actively copied during RB replication. To this end, we used primers targeting a previously characterized, single-copy chromosomal gene *tufA* encoding translation initiation factor EF-Tu (33, 34). With similar infection rates in HeLa cells, the *C. trachomatis* L2/*topA*-kd and L2/Nt exhibited a similar increase (~2.1-fold) in gDNA amounts at 15 h pi under dCas12-inducing conditions (Fig. 4b), perhaps reflecting a slight acceleration of RB multiplication by aTC addition. However, the gDNA amounts were unchanged at 24 h pi. These results indicate significant levels of RB accumulation in both L2/*topA*-kd and L2/Nt. The lack of a meaningful difference in gDNA amounts between L2/*topA*-kd and L2/Nt indicates that both strains progress through early developmental stages without issue, further supporting that dCas12 induction alone was harmless for *C. trachomatis*.

It is known that the chlamydial developmental cycle is coupled to temporal gene expression (28, 35, 36). If perturbation of EB formation, but not RB replication, occurs, then *de novo* synthesis of early gene products is unaffected, whereas late gene expression is diminished. We tested whether we could find evidence of impaired EB formation (i.e., secondary differentiation). Nucleic acid samples were collected from HeLa cells infected with the L2/*topA*-kd or L2/Nt at 15 and 24 h pi in the absence or presence of aTC. Expression of four genetic markers specific to *Chlamydia* developmental stages was examined using RT-qPCR: (i) *incD* encoding an early inclusion membrane protein, IncD, which may be needed to establish the inclusion niche (37); (ii) *euo* encoding EUO that can bind to and repress some late promoters (38, 39); (iii) *omcB* encoding an EB-related 60-kDa cysteine-rich outer membrane protein OmcB; and (iv) *hctB* encoding HctB needed for chromatin condensation (40). The transcript levels of these genes were normalized to the gDNA amounts that were concurrently measured with the same primer pair for comparison. Regardless of whether aTC was added, there were less than ~1.5-fold changes in transcript levels of *incD* and *euo* in L2/*topA*-kd and L2/Nt at both 15 and 24 h pi (Fig. 4; Fig. S4). In contrast, adding aTC decreased the levels of *omcB* and *hctB* by ~50% in L2/*topA*-kd at 24 h pi. There were abundant *omcB* and *hctB* transcripts in L2/Nt. The disparity in OmcB protein levels between L2/*topA*-kd and L2/Nt was confirmed by IFA. OmcB is considered to be an EB-related marker because it provides integrity to the outer envelope via disulfide crosslinks with OmcA and MOMP (41). The fluorescence intensity of OmcB labeling associated with the inclusions appeared lower upon *topA* repression than mock repression in L2/*topA*-kd (Fig. 4d), and it was unchanged in L2/Nt (Fig. 4e). Collectively, changes in the chlamydial stage-specific gene expression profile combined with the growth curve data (Fig. 2) imply that *topA* knockdown disrupts secondary differentiation from RB to EB. Conversely, the gDNA measurements suggest that RB accumulation is unaffected.

### Overexpressing *topA* has varying effects on the growth of *C. trachomatis*

**Fig 5:**
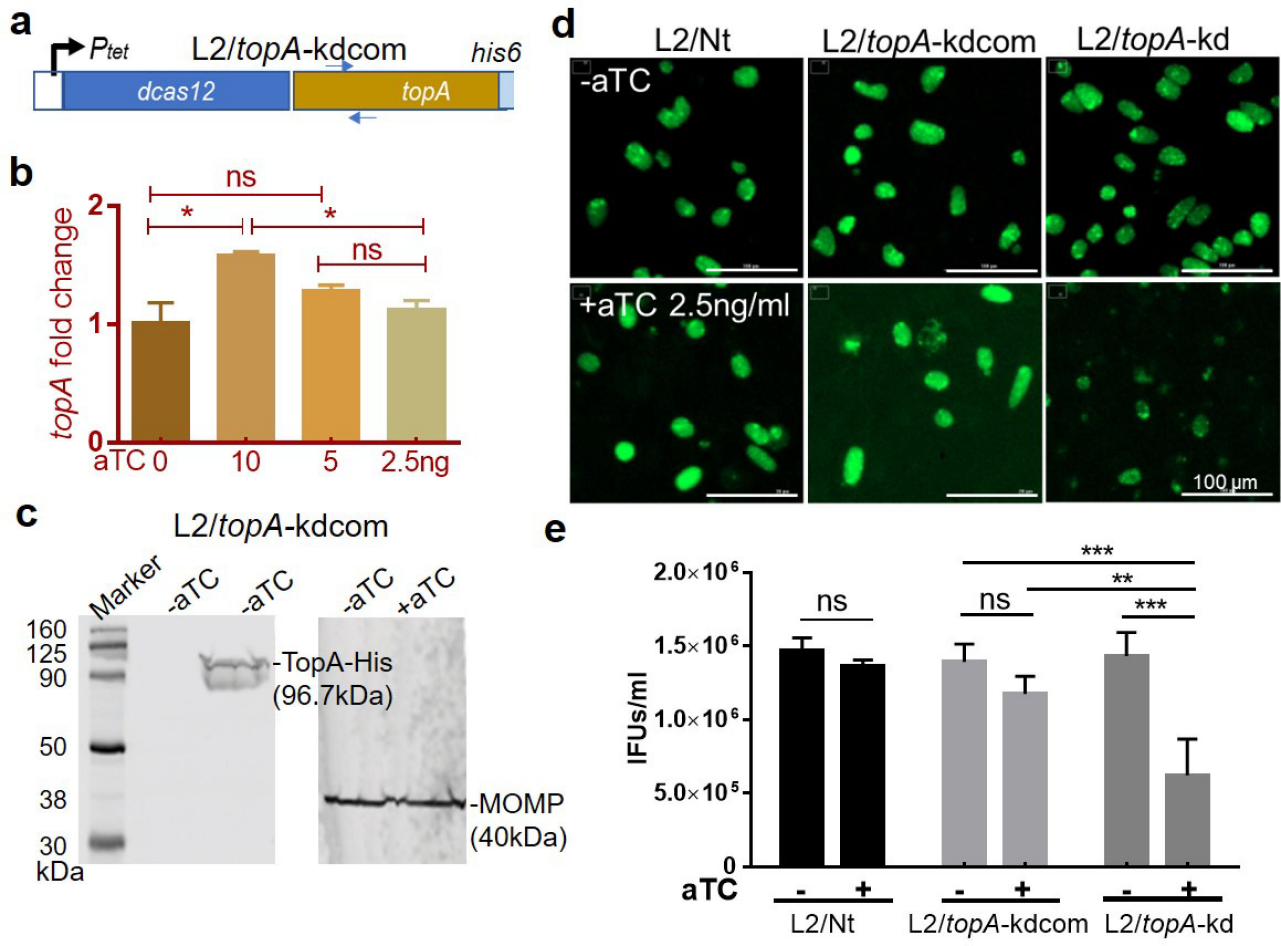
Effect of aTC addition on the growth of *C. trachomatis* L2/*topA*-kdcom. (a) Schematic map of the expression vector containing *topA-His*6 that is co-regulated with *dcas12* by aTC-inducible P*_tet_* and *topA*-specific crRNA (not shown). (b) RT-qPCR analysis of *topA* transcripts in *C. trachomatis* L2/*topA*-kdcom. Nucleic acid samples from HeLa cells infected with L2/*topA*-kdcom were collected at 24h pi. The locations of primers used to detect *topA* from the cDNA samples are shown in panel a and detailed in Table S2. (c) Immunoblot displays the inducible expression of TopA-His6 in *C. trachomatis*. TopA-His6 protein from the lysates of *C. trachomatis*-infected cells was isolated by 10% SDS-PAGE for immunoblot with antibody against His6 or L2 MOMP. (d) Representative live-cell images of HeLa cells infected with *C. trachomatis* strains as indicated. HeLa cells with infection of *C. trachomatis* L2/*topA*-kdcom or the control strains, L2/Nt and L2/*topA*-kd, were cultured in RPMI-10 lacking (−aTC) or containing an optimal concentration of aTC (2.5 ng/mL). After 24 h pi, infected cells were imaged in combination with green fluorescence and bright light detection using Cytation1. Scale bar = 100 µm. (also see Fig. **S8**) (e) Enumeration of EBs using IFU assay. The infected cells and culture supernatants were collected at 40 h pi and used to infect a fresh HeLa cell monolayer for enumeration of recoverable IFU. Data are representative of those from an experiment performed in triplicate for each condition and presented as IFUs (mean ± SD). Three independent experiments were performed with similar results. Statistical significance was determined by one-way ANOVA followed by Tukey’s post hoc test. *P ≤ 0.05, **P ≤ 0.01, ***P ≤ 0.001.

To confirm that the impaired growth phenotype observed is due to *topA* knockdown, we created a strain to complement TopA expression during knockdown as demonstrated previously for other targets (22, 23, 42, 43). The full-length *topA* gene with a six-histidine (his6)-tag was cloned and transcriptionally fused 3′ to the *dCas12* in pBOMBL12CRia(*topA*)::L2, resulting in pBOMBL12CRia-*topA*_6xH(*topA*)::L2 (Fig. 5a; Fig. S5). In this vector, the *topA-his6*, as a source of the *topA*, is co-expressed with the *dCas12* under the control of the aTC-induced P*_tet_* promoter. Because the dCas12 in combination with the *topA*-specific crRNA targets the promoter region of *topA* on the chromosome and causes growth defects*,* the functional protein product of *topA-his6* should correct the CRISPRi-mediated growth defect of *topA* knockdown.

To determine if *topA-his6* could be induced, *C. trachomatis* was transformed with pBOMBL12CRia-*topA*_6xH(*topA*)::L2, resulting in strain L2/*topA*-kdcom, which was then used to infect HeLa cells. We confirmed the inducible expression of *topA* using RT-qPCR (Fig. 5b). As we did not have access to specific anti-sera against *C. trachomatis* TopA, antibody against His6 was used to examine TopA-His6 protein by immunoblotting analyses of extracts from cells infected with L2/*topA*-kdcom. We found an immunoreactive ~98-kDa band in the presence of aTC (Fig. 5c). An additional ~85-kDa immunoreactive band was seen. The nature of this band is uncertain, but we speculate that it could represent translation of an aborted *topA* transcript or an N-terminal degradation product of TopA-His6. The identity of the band corresponding to the full-length TopA-His6 protein was further validated by mass spectrometry (Fig. S6). Additionally, IFA was used to detect dCas12 expression (Fig. S7), indicating the induction of TopA-His6 did not impair dCas12 expression in the L2/*topA*-kdcom strain.

Given the co-induction of dCas12 and TopA-His6 in L2/*topA*-kdcom, we speculated a balance between *topA* repression and *topA-his6* overexpression would occur as a function of the aTC inducer concentration due to the copy number difference between the chromosomal target and the plasmid-encoded complementing allele. To adequately establish the optimal conditions for *topA* complementation, *C. trachomatis* growth was assessed by measuring the EB yields and GFP MFI in the presence of increasing amounts of aTC (Fig. S8). *C. trachomatis* L2/*topA*-kdcom displayed a different phenotype from that of L2/*topA*-kd or L2/Nt in response to aTC (Fig. 5d and e; Fig. S8). The addition of aTC decreased EB yields in L2/*topA*-kd due to *topA* repression, whereas the addition of aTC to L2/Nt did not impact EB yields, as expected. Under the same conditions, L2/*topA*-kdcom produced more EBs and exhibited stronger GFP signal than L2/*topA*-kd. Interestingly, the larger the amount of aTC added, the smaller the improvement was observed in L2/*topA*-kdcom growth. These results likely reflect the combination of *topA* repression and *topA-his6* overexpression as both are induced by aTC. TopA-His6’s real action in correction of the growth defect might be masked at a higher concentration of aTC under this condition. Nevertheless, the appropriate *topA* induction increased EB and GFP levels comparable to wild-type (WT) levels when aTC was used at ≤5 ng/mL. The detection of EB-related OmcB also indicated increased EB accumulation in *C. trachomatis* L2/*topA*-kdcom (Fig. S9)

**Fig 6:**
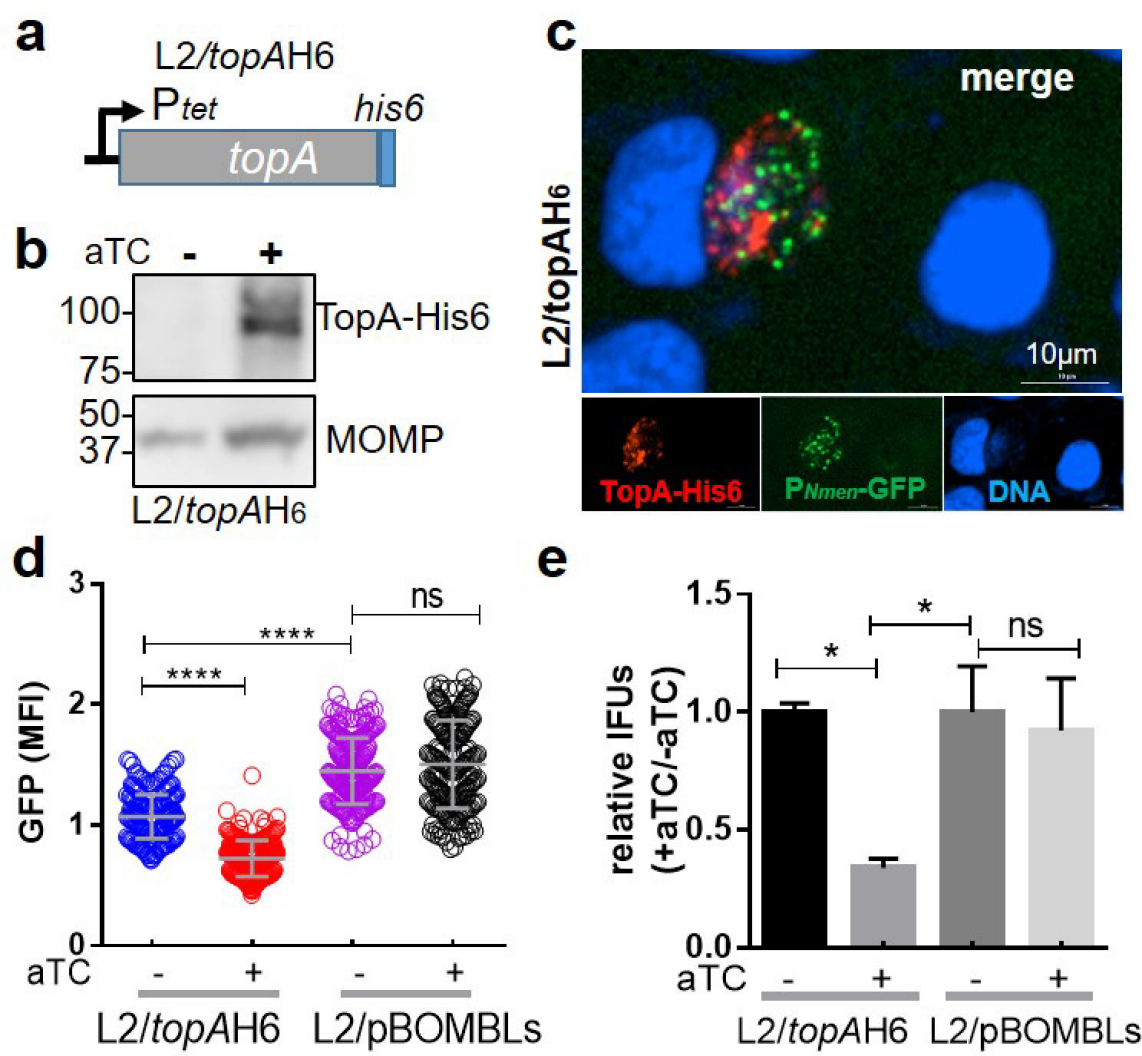
Overexpression of *topA-His6* has detrimental effects on *C. trachomatis*. (a) Schematic map of the expression vector pBOMBLs-*topA*His6, in which *topA-His_6_* is under the control of aTC-inducible P*_tet_*. (b) Immunoblot for TopA-His6. The proteins from lysates of *C. trachomatis* L2/*topA*H6-infected HeLa cells harvested at 40 h pi were separated by SDS-PAGE. Blot was stained with antibody against His6 (upper panel) or L2 MOMP (lower panel). (c) Immunofluorescent micrographs of *C. trachomatis* L2/*topA*H6 in the presence of aTC (5 ng/mL) added at 0 h pi. Cells were fixed at 24 h pi and used for IFA with mouse anti-His6 antibody and visualized with Alexa fluor 568-conjugated goat anti-mouse IgG. Scale bar = 10 µm. (d) Evaluation of P*_Nmen_*-GFP levels. Live-cell images were acquired at 24 h pi. Individual chlamydial inclusions equal to 257 ± 52 per condition were measured. Values were obtained from triplicate results in a representative experiment and are shown as mean ± SD. At least three independent experiments were performed. (e) Enumeration of EB yields. *C. trachomatis* L2/*topA*H6 or L2/pBOMBLs-infected cells were cultured for 24 h in the absence (−aTC) or presence (+aTC, at 5 ng/mL) of aTC. Triplicate results in a representative experiment are shown as mean ± SD. The data are presented as the ratio of IFUs from the aTC-exposed sample to that from the aTC-unexposed sample, which is set at 1. At least three independent experiments were performed. Statistical significance was determined by one-way ANOVA followed by Tukey’s post hoc test. *P ≤ 0.05, ****P ≤ 0.0001. ns, no significance.

We hypothesized that the reduced EB accumulation in L2/*topA*-kdcom at higher aTC concentrations was due to adverse effects of overexpressed TopA in comparison to the uninduced control; in other words, the complemented TopA levels exceed the normal levels of TopA under non-inducing conditions. To determine the effect of TopA overexpression alone, *C. trachomatis* L2/*topA*H6 was created by transforming with pBOMBLs-*topA*his6 containing the P*_tet_*-driven *topA-his6* and lacking CRISPRi elements (i.e., *dcas12* and crRNA) (Fig. S10). HeLa cells were infected with strain L2/*topA*H6 or the control strain, L2/pBOMBLs. The GFP MFI and EB levels were evaluated. Immunoblotting and IFA confirmed aTC-dependent induction of TopA-His6 expression along with a minor increase in MOMP level (Fig. 6c), the pattern of which differed from that observed in L2/*topA*-kdcom (Fig. 5c). In contrast, adding aTC (at 5 ng/mL) decreased GFP signal and the EB yield in L2/*topA*H6, while there was no change in L2/pBOMBLs under these conditions. These results suggest that excessive TopA protein or activity is detrimental to *Chlamydia*, consistent with the varying effects on *C. trachomatis* L2/*topA*-kdcom, depending on aTC dose used. The detrimental effects of an increased amount of TopA on bacterial growth have been reported in *E. coli* and *Salmonella* Typhimurium, both of which displayed reduced viability under these conditions (44).

Genetic complementation with TopA-His6 during *topA* knockdown (in strain L2/*topA*kdcom) at an appropriate time and condition can conditionally improve the EB production, indicating that *topA* knockdown is responsible for the incomplete developmental cycle of L2/*topA*-kd. These data, combined with the results for L2/*topA*H6 overexpressing *topA* and lacking CRISPRi elements, demonstrate the requirement for a suitable TopA level and activity to support the developmental cycle of *C. trachomatis*.

### Targeted *topA* knockdown affects expression of DNA gyrase genes

Our data thus far reveal that CRISPRi-mediated *topA* repression has a profound impact on the *C. trachomatis* developmental cycle along with selectively downregulated transcription of chromosomal genes (i.e., late genes, *omcB* and *hctB*) and the plasmid-encoded P*_Nmen_-gfp*. These data are in line with previous studies in *E. coli* that suggested a key role of TopA in controlling transcription (2, 44–46). Previously, Orillard and Tan (21) proposed a feedback regulatory mechanism of Topo genes in *C. trachomatis*. We asked whether *topA* repression altered transcription of gyrase encoded genes (*gyrA*/*gyrB*) and TopoIV encoded genes (*parE*/*parC*); both enzymes are targets of moxifloxacin. *C. trachomatis gyrB* and *gyrA* are adjacent and located downstream of *ctl0443*/*ct191* encoding a hypothetical protein. These genes are co-regulated by a supercoiling sensitive promoter upstream of *ctl0443/ct191* (21). Similarly, *parE* and *parC* are adjacent genes that are controlled by a supercoiling sensitive promoter. These polycistronic mRNAs were assessed at 24 h pi using RT-qPCR during *topA* knockdown (in L2/*topA*-kd) or complementation (in L2/*topA*-kdcom). In the control experiments, L2/Nt was used.

**Fig 7:**
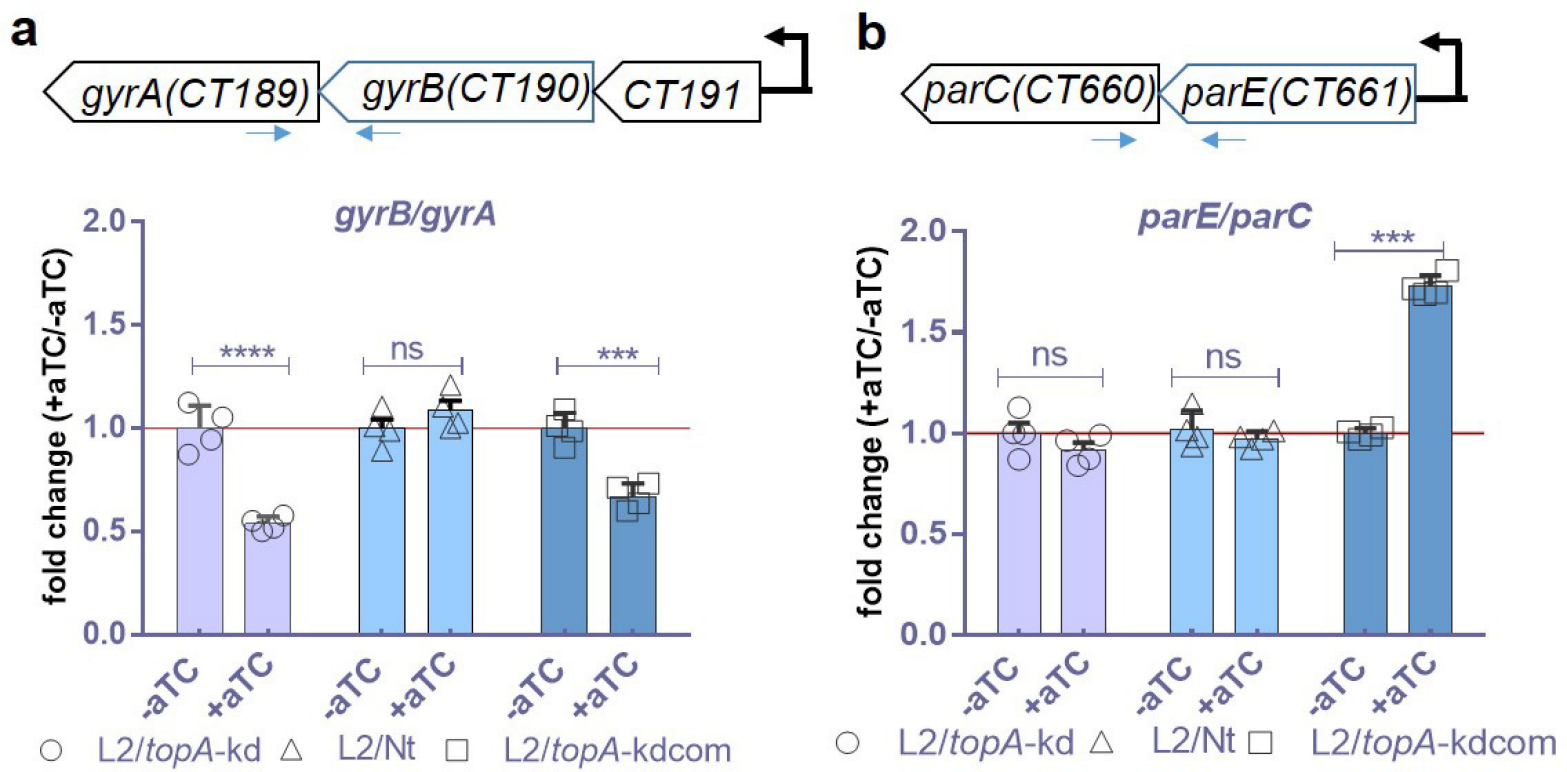
Effects of CRISPRi-induced *topA* knockdown on expression of DNA gyrase genes and topoIV genes in *C. trachomatis*. (a) Schematic map of *gyrB/gyrA* operons in *C. trachomatis* and detection of their transcript products using RT-qPCR. (b) Schematic map of *parE/parC* in *C. trachomatis* and detection of their transcript products using RT-qPCR. HeLa cells infected with *C. trachomatis* L2/*topA*-kd, L2/Nt, or L2/*topA*-kdcom were grown in the presence or absence of aTC (at 5 ng/mL) and harvested at 24 h pi for total RNA preparation and then cDNA synthesis. The location of primer pairs used for RT-qPCR analysis is shown. Results were obtained from two independent experiments performed in duplicate for each condition. The graph indicates mean ± SD of the ratio of transcripts from the aTC-exposed sample to that from the aTC-unexposed sample, which is set at 1 as shown by a red line. Statistical significance was determined by one-way ANOVA followed by Tukey’s post hoc test. ***P ≤ 0.001, ****P ≤ 0.000. ns, no significance.

The targeted manipulation of *topA* was detectable at harvest time following addition of aTC (at 5 ng/mL); i.e., *topA* transcripts were reduced in L2/*topA*-kd, while *topA* transcripts were induced in L2/*topA*kdcom. Whereas the *gyrB*/*gyrA* transcripts were unchanged in L2/Nt, they were decreased in L2/*topA*-kd and L2/*topA*-kdcom (Fig. 7). These results are unsurprising as the different Topos may compensate for a defect in one enzyme by varying expression of another as reported in *E. coli* and *S. typhimurium* (8, 47). Unlike the results for *gyrB*/*gyrA*, expression of *parE*/*parC* was unchanged in L2/*topA*-kd and in L2/Nt, but it was increased (1.7-fold) in L2/*topA*-kdcom. Since aTC induced CRISPRi-mediated *topA* repression in L2/*topA*-kd, these observations imply that the proportion of gyrase synthesis was regulated in *C. trachomatis* in response to *topA* repression under our tested conditions. Reduced *gyrB*/*gyrA* expression in L2/*topA*-kd is not due to mutation in the promoter region of *ctl0443*/*ct191* as analyzed by PCR and DNA sequencing analysis (data not shown). For L2/*topA*-kdcom, in which *topA-his6* was induced and the chromosomal *topA* was repressed by adding aTC, 1.5-fold increase in TopoIV (i.e., *parE*/*parC*) transcripts and 0.3-fold decreased gyrase (i.e., *gyrB*/*gyrA*) transcripts were measured (Fig. 7). These transcripts were unchanged in the control L2/Nt strain. It is unlikely that the CRISPRi constructs used have off-target effects based on whole genomic sequence analysis. Therefore, the observed changes in expression of gyrase genes (in L2/*topA*-kd) or the TopoIV genes (in L2/*topA*-kdcom) suggest compensatory responses expected from homeostatic regulation. These data support the notion that Topo activity is carefully balanced in *C. trachomatis* to, perhaps, maintain appropriate levels of DNA supercoiling.

### Targeted *topA* knockdown affects the response of *C. trachomatis* to moxifloxacin

A low level of tolerance to quinolones was associated with reduced expression of quinolone targets, gyrase and/or TopoIV, in bacteria (8, 48). Having found decreased transcription of *gyrB*/*gyrA* in L2/*topA*-kd following repression of *topA*, we postulated that the sensitivity of *C. trachomatis* to Mox might be altered. To test this hypothesis, the minimal inhibitory concentration (MIC) of Mox was initially determined with *C. trachomatis* reference strain, L2/434/Bu, in HeLa cells. The concentrations of Mox that resulted in decreases in IFUs to 50% and 99% of the unexposed culture were 4.5 and 50.0 ng/mL, respectively, using IFU assay and IFA in combination (Fig. S11). The MIC was determined as 100 ng/mL, where no IFUs were detected in subculture. Similar results were obtained in *C. trachomatis* strains L2/Nt, L2/*topA*-kd, and L2/*topA*-kdcom in the absence of aTC addition.

We next sought to determine if *topA* knockdown influenced the sensitivity of *C. trachomatis* to Mox. A sub-MIC concentration of Mox (5 ng/mL = 1/20 MIC) was used because we were interested in phenotypic changes in live *C. trachomatis* and a high Mox dose could be lethal. The phenotypes of *C. trachomatis* L2/*topA*-kd, L2/Nt, and L2/*topA*-kdcom in HeLa cells were assessed in the absence of Mox, presence of Mox alone, or aTC + Mox by measuring the inclusion size, GFP MFI, IFUs, and gDNA contents.

**Fig 8:**
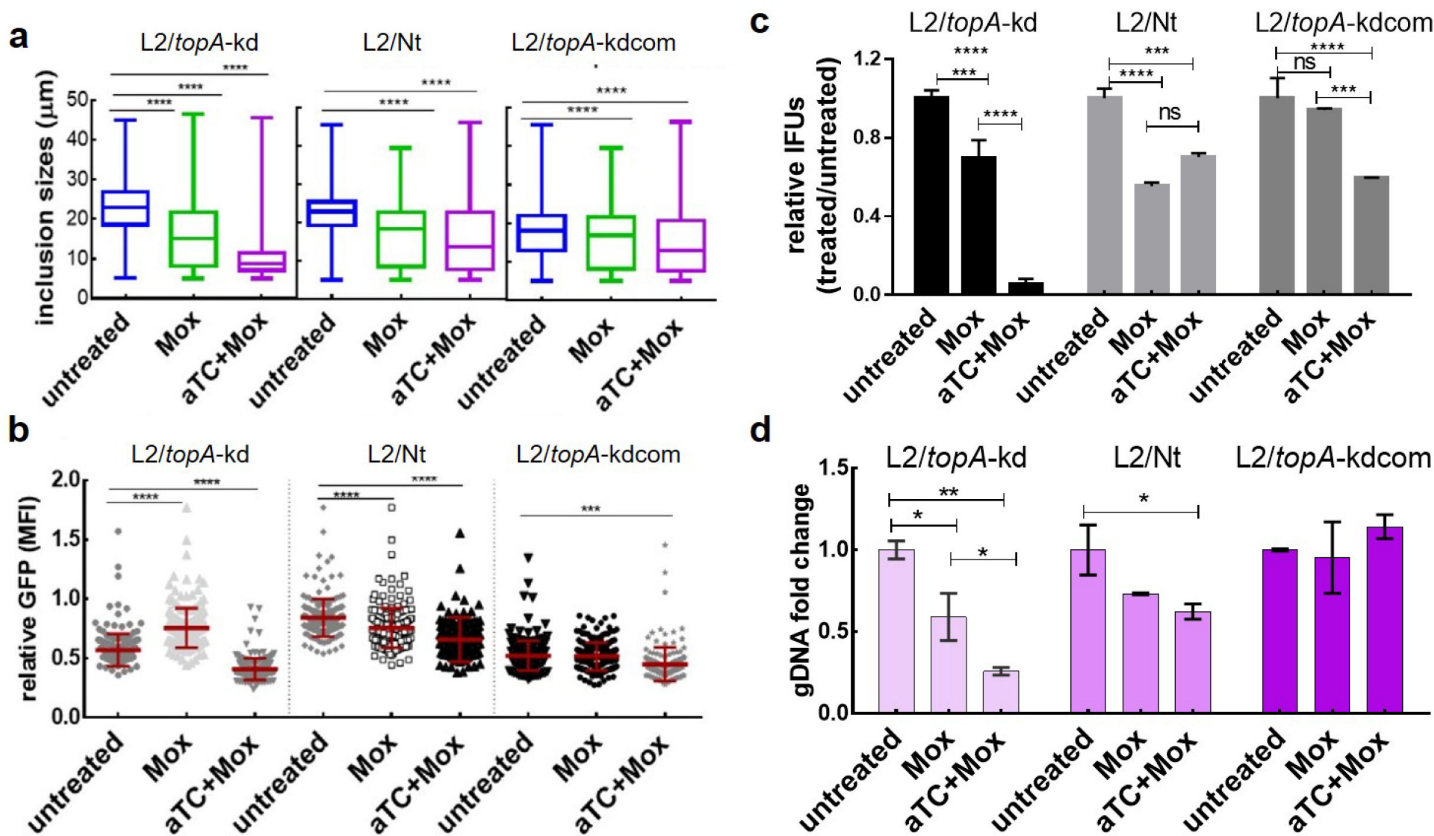
Analysis of the response of *C. trachomatis* to moxifloxacin. (**a** and b) Comparison of chlamydial inclusion sizes (a) and the GFP MFI (b) of L2/*topA*-kd to those of L2/Nt and L2/*topA*-kdcom. HeLa cells were infected by L2/*topA*-kd, L2/Nt, or L2/*topA*-kdcom, cultured in the absence or presence of Mox (5 ng/mL) or both aTC (5 ng/mL) and Mox (5 ng/mL), and imaged at 42 h pi. The individual chlamydial inclusions equal to 198 ± 14 were analyzed. (c) Enumeration of EB yields. *C. trachomatis*-infected cells were harvested at 40 h pi and used for IFU assay. IFU values from triplicate results in a representative experiment are presented as mean ± SD. Data are presented as the ratio of IFUs from treated samples to that from untreated sample, which is set at 1. Three independent experiments were performed. (d) Analysis of *C. trachomatis* gDNA in the presence or absence of aTC using real-time qPCR. Values are presented as the ratio of chlamydial gDNA copy numbers per nanogram DNA in treated sample to that in the untreated sample, which is set at 1. Triplicate results in a representative experiment are shown. Three independent experiments were performed. Statistical significance was determined by two-way ANOVA followed by Tukey’s post hoc test. *P ≤ 0.05, **P ≤ 0.01, ***P ≤ 0.001*,* ****P < 0.0001.

The addition of Mox or aTC + Mox decreased inclusion sizes in all strains tested at 40 h pi (Fig. 8). Interestingly, Mox or aTC + Mox reduced GFP MFI in L2/Nt and L2/*topA*-kdcom, while aTC + Mox, but not Mox alone, weakened the GFP signal in L2/*topA*-kd. Consistent with the Mox-induced decrease in inclusion sizes, EBs in L2/*topA*-kd and L2/Nt were ~30% and ~50% less than the untreated controls, respectively, at 40 h pi. Conversely, there was no change in L2/*topA*-kdcom. With aTC + Mox in the medium, EB yields were decreased in all strains with the most prominent reduction in L2/*topA*-kd (~90% less) as analyzed by IFU assay. In support of the IFU data, decreased chlamydial gDNA contents were measured at 24 h pi for L2/*topA*-kd and L2/Nt in the aTC + Mox condition. Interestingly, the gDNA content in L2/*topA*-kdcom was unchanged by either Mox or aTC + Mox. We confirmed the induction of dCas12 and TopA-His6 in L2/*topA*-kdcom in the presence of aTC + Mox (Fig. S12). The unchanged gDNA contents, in contrast to the reduced EBs in L2/*topA*-kdcom, raise several possibilities, including continued production of RBs without progression through the late developmental cycle, the induction of persistent forms that are viable but non-infectious, or, potentially, dead bacteria.

Together, these results imply disparities in the responses of *C. trachomatis* to the sub-MIC of Mox among strains L2/*topA*-kd, L2/Nt, and L2/*topA*-kd, suggesting that the levels of *topA* expression and/or its potentially subsequent effects on gyrase and/or TopoIV likely influence the chlamydial response to *moxifloxacin*.

## DISCUSSION

Topos have been extensively studied since the first discovery of bacterial TopoI in 1971 (49). These enzymes are recognized targets for drug development (6–9). However, the understanding of Topos and their relevance to *C. trachomatis* development has progressed more slowly, hampered in large part by the lack, until recently, of tractable genetic techniques for *Chlamydia*. The recent development of CRISPRi as a genetic tool to inducibly repress transcription in *Chlamydia* allowed us to demonstrate an indispensable role of TopA in controlling chlamydial developmental cycle progression.

### Utility of CRISPRi system in *C. trachomatis*

Studying essential genes, such as Topo encoded genes, is challenging because conventional gene disruption strategies lead to lethality. Likely due to the essentiality of TopA, *topA* depletion or insertion mutants frequently carry compensatory mutations in gyrase genes or other related gene(s) in *E. coli* (9, 50). CRISPRi has provided inducible knockdown of gene expression and enabled genetic approaches to study essential gene function in several bacterial pathogens (51–53). Only a handful of studies have reported the use of CRISPRi in intracellular bacteria (22, 54, 55). We have demonstrated successful knockdown of *topA* in *C. trachomatis* using a plasmid-based CRISPRi system that relies on the combination of inducible dCas12 and a *topA*-specific crRNA (Fig. 1 through 3). The incorporation of P*_Nmen_*-GFP as a reporter facilitates monitoring the influence of *topA* repression on *C. trachomatis*. We also show the effects of dose, time, and duration of aTC addition on the efficiency and degree of CRISPRi-mediated *topA* repression. These results reveal the unique *C. trachomatis* developmental cycle that is affected by TopA activity (Fig. 4a through d ). Since RBs are highly active in macromolecular synthesis and metabolically different from EBs (32), it is unsurprising that the growth rate and physiological state of *C. trachomatis* play a role in determination of dCas12 induction as observed in the current studies. It is also likely that the distinct chlamydial forms respond to aTC and induce dCas12 differently, a factor that should be taken into consideration when designing experiments and interpreting results.

An important issue when using CRISPRi for gene knockdown is its specificity or potential off-target effects. The chlamydial genome is small (~1 Mbp). This, in and of itself, minimizes the risk of off-target effects. In general, guide RNAs with up to three or four mismatches can result in off-target effects but also require a PAM sequence. Off-target effects also require the correct DNA strand to be targeted in the intergenic or 5′ region of the gene coding region. Our design of *topA*-specific crRNA had a minimum of seven mismatches (14- out of 21-bp homology) to other chromosomal sequences and, importantly, no PAM sequence, thus, off-target effects from the *topA* crRNA are unlikely. In fact, three independent observations indicate targeted *topA* repression is specifically mediated by CRISPRi in our study. First, our genome sequence analysis shows, besides the *topA* promoter region, no sequence similarity to the *topA*-targeting crRNA in *C. trachomatis*. Further, no homology between the crRNA and any plasmid gene was identified. Second, no reduction of *topA* transcription was observed in the control strain containing a non-targeting crRNA that has no homology to chlamydial sequences. Finally, the negative effects of *topA* repression on *C. trachomatis* were reversed by complementation with a plasmid-encoded *topA* when expressed at the appropriate level and induction time in L2/*topA*-kdcom (Fig. 5a through e; Fig. S8).

### The role of TopA in the chlamydial developmental cycle

**Fig 9:**
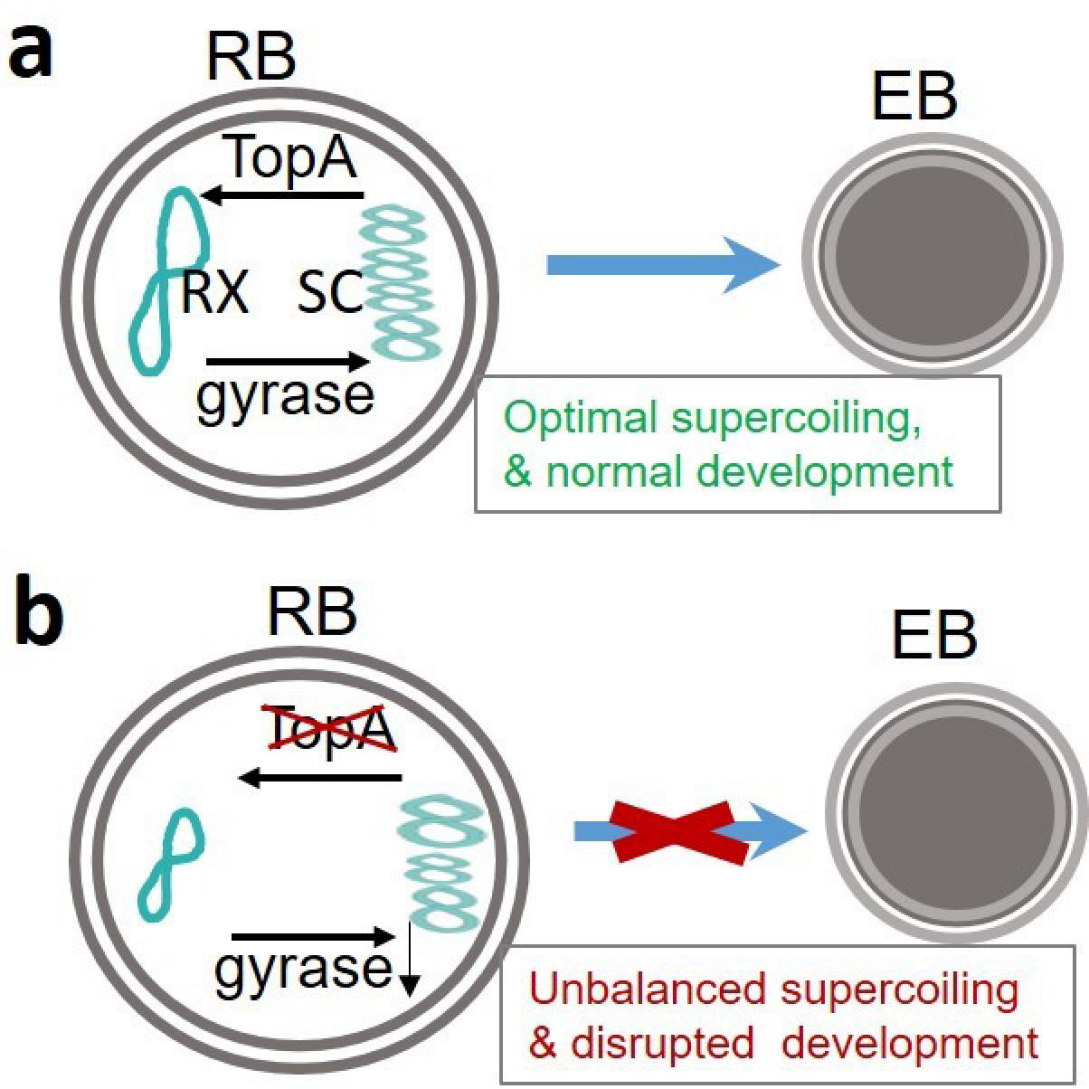
Schematic highlighting the role of TopA in *C. trachomatis* developmental cycle. (a) In wild-type *C. trachomatis*, optimal supercoiling levels during chlamydial developmental cycle progression is primarily maintained by the joint action of *topA* that relaxes DNA supercoiling (RX) and gyrase that induces negative supercoiling (SC).(b) When *topA* is repressed, the DNA supercoiling is predicted to increase, resulting in changes in expression of supercoiling-sensitive genes (e.g., *gyrB/gyrA* and *omcB*), thus perturbing chlamydial development. Our data indicate that the carefully balanced activities of TopA and gyrase contribute to the completion of the chlamydial developmental cycle.

Our results suggest a model where TopA and gyrase act together to support chlamydial development (Fig. 9). Since TopA is an essential enzyme that primarily acts to relax negative supercoiling during transcription, we evaluated the effects of knocking down expression of this enzyme on the *C. trachomatis* developmental cycle by assessing overall EB yields, one-step growth curves, and inclusion growth and expansion (Fig. 1 through 4; Fig. S3). Changes in P*_Nmen_*-GFP levels also allowed for the sensitive detection of *topA* repression-mediated impacts. The data, using multiple methods to detect differences in development between the WT strain and strains complemented or not for *topA* knockdown, indicate the requirement of a proper level of TopA to complete the chlamydial developmental cycle. The data showing *topA* knockdown has no effect on RB or gDNA accumulation (Fig. 4) suggest that reduced TopA activity does not significantly impair the EB-to-RB transition or RB replication. However, and conversely, TopA activity is crucial during the process of RB-to-EB differentiation. Interestingly, we found that the developmental progression of the L2/*topA*-kdcom strain was slowed when the plasmid-encoded *topA-his6* complementing allele was highly expressed (Fig. 5a through e; Fig. S8). We evaluated the possibility that the detrimental effect of an increased amount of TopA might be associated with this suboptimal growth. Indeed, with lower *topA-his6* induction, L2/*topA*-kdcom displayed improved developmental progression as evidenced by increased EB yields and EB-related markers. The suboptimal growth effect was also observed when TopA-His6 is overexpressed in the absence of the CRISPRi system (i.e., L2/*topA*H6 strain) (Fig. 6a through d). These data give further support that *C. trachomatis* is sensitive to altered TopA levels.

It is worth noting that the chlamydial inclusions in the complemented knockdown or clean overexpressing strains tend to be smaller than those of the respective control strains even without the addition of aTC. We cannot exclude that a trace amount of TopA-His_6_ is expressed under this condition, although we also note our inability to detect TopA-His_6_ using IFA and immunoblotting analysis. The potential leaky P*_tet_* activity in *C. trachomatis* culture has been reported previously (56–58). Future studies may require the development of a more tightly controlled expression system (56, 57) for complementation or overexpression of potential “toxic” proteins in *C. trachomatis*. We are currently working on such systems.

### The role for TopA in gene regulation of *C. trachomatis*

DNA topology affects regulation of gene expression both locally and globally at the transcription level. Recent genome-wide transcriptomic data suggest that elaborate mechanisms are employed by bacteria to coordinate transcription rates and Topo activity to adjust supercoiling levels in the promoter regions of differentially expressed genes (44, 46, 59). Bacteria maintain supercoiling homeostasis by regulating transcription of their topo genes. Although the precise role of topos in global transcription in *C. trachomatis* remains to be determined, previous studies indicated differences in plasmid DNA supercoiling during the chlamydial developmental cycle (13, 17, 18, 20) and that several putative promoters and respective genes appeared to be more “supercoiling sensitive” than others (18, 20, 21). Three promoters of chlamydial Topo genes are reported to act in a supercoiling-dependent manner. Complementing and extending these findings, our data demonstrate that the transcript levels of gyrase genes were decreased following *topA* repression, while the levels of TopoIV were unchanged in *C. trachomatis*. We did not have access to specific antibodies against the subunits of chlamydial gyrase and TopoIV and thus did not measure their levels directly. However, the decreased levels of *gyrA/gyrB* transcripts suggest that the levels of the gyrase were likely reduced. In *E. coli*, TopA relaxes negatively supercoiled DNA and has been shown to sustain the steady-state level of supercoiling by balancing the activity of DNA gyrase (3, 5). Since the main functions of Topos are to prevent excessive supercoiling that is deleterious, we speculate that, if *topA* is knocked down, negative supercoiling will increase. Subsequent decreases in gyrase expression likely occur, perhaps, to balance the supercoiling levels for maintaining *Chlamydia* viability (Fig. 9). Although the current study did not directly show increased DNA supercoiling in *Chlamydia* with *topA* knockdown, increases in general DNA supercoiling were observed upon Topo A inhibition in *E. coli* (5) and *S. pneumoniae* (60), and the opposite was observed by inhibition of gyrase using novobiocin. Our data recapitulate the relationship between TopA and the type II Topos and suggest the contribution of opposing catalytic activities of TopA (to relax) and DNA gyrase (to supercoil) to transcription of topo genes in *C. trachomatis*.

Our results provide strong evidence that the P*_Nmen_-gfp* from plasmid is affected by Topo homeostasis. Targeted *topA* knockdown decreased overall levels of GFP in *C. trachomatis*, but the mechanism underlying this is unclear. We speculate two different mechanisms that might be associated with changes in GFP expression in *C. trachomatis* L2/*topA*-kd. First, transcription activity of the P*_Nmen_* is directly modulated by *topA* repression that alters the supercoiling level. Second, *topA* repression affects DNA replication and reduces plasmid copy numbers, in turn decreasing P*_Nmen_-gfp* expression. Interestingly, inhibition of gyrase/TopoIV at a low dose of moxifloxacin alone increases GFP levels in L2/*topA*-kd, suggesting an opposing effect of the gyrase on P*_Nmen_-gfp*. Thus, changes in P*_Nmen_-gfp* levels appear to be a sensitive indication for balanced/imbalanced Topo activity in *C. trachomatis*.

### Response of *C. trachomatis* to moxifloxacin

To evaluate the outcome linked to *topA* repression, we examined whether *topA* repression influenced the response of *C. trachomatis* to moxifloxacin. Although TopA is not the target of moxifloxacin, inhibition or overexpression of *topA* changes the expression levels of gyrase or TopoIV genes (Fig. 7). Unlike novobiocin that inactivates ATPase activity of the GyrB subunit of gyrase, moxifloxacin (Mox) dually inhibit activities of gyrase GyrA and TopoIV and can form a poisonous Topo-quinolone-DNA complex that eventually breaks double-stranded DNA leading to bacterial death (6, 7). Because aminocoumarin and quinolones are potential inducers of SOS-related stress responses (61), changes induced by antibiotic exposure may reflect effects of both supercoiling and that of the supercoiling-independent stress responses. These mechanistically different contributors are hard to distinguish. However, it should be noted that *Chlamydia* lacks a conventional SOS response. Nonetheless, we used a sub-MIC concentration of moxifloxacin to determine the impacts of *topA* repression on moxifloxacin sensitivity. With the strain L2/*topA*-kd, a hypersensitivity to Mox was linked to *topA* knockdown. This result is unexpected, as, based on observations from other bacteria, decreased levels of the drug target (i.e., gyrase) may reduce formation of poisonous complexes and thus weaken drug action, leading to quinolone tolerance. Moxifloxacin had different effects on the strain L2/*topA*-kdcom, depending on the degree of *topA* induction. While L2/*topA*-kdcom displayed a low level of tolerance to moxifloxacin, its growth was inhibited under *topA* knockdown or induction conditions. Although the mechanisms remain to be determined, slowly growing *C. trachomatis* has been shown to sense antibiotics irrespective of what class of drug is used (62, 63). The aberrant *C. trachomatis* induced by *topA* overproduction or repression might provide a partial explanation for the differences in the responses to Mox between L2/Nt, L2/*topA*-kd, and L2/*topA*-kdcom. There might also be a proportion of dead *Chlamydia* in the presence of Mox. However, most bacteria remained viable under our testing conditions because they resume normal growth after aTC + Mox containing medium was replaced with normal medium starting at 24 h pi (L. Shen et al., unpublished observation). The significance of the moxifloxacin sensitivity studies is unclear, and further study is necessary to understand the mechanisms underlying the paradoxical sensitivity of the L2/*topA-*kd to Mox.

### Summary

We have leveraged novel approaches in *Chlamydia* to demonstrate for the first time that carefully balanced TopA activity is required for the completion of the *C. trachomatis* developmental cycle. Despite the evidence that changes in *topA* levels affect transcription of selected *C. trachomatis* stage-expressed genes, the overall influence of TopA activity on the physiology of *C. trachomatis* remains to be determined. Future studies will attempt to understand the precise role of TopA in global gene regulation, DNA replication, and recombination.

## MATERIALS AND METHODS

### Reagents and antibodies

Antibiotics and dimethyl sulfoxide (DMSO) were purchased from MilliporeSigma (St. Louis, MO, USA). Moxifloxacin stock solution was dissolved in 100% DMSO at 10 mg/mL. In all experiments, the moxifloxacin stock was diluted in the corresponding culture medium, and controls lacking moxifloxacin were performed using an equal percentage of DMSO. Digest restriction enzymes, alkaline phosphatase, and DNA Phusion polymerase were purchased from Thermo Fisher (Waltham, MA). The following primary antibodies were used to detect proteins of interests: (i) a mouse monoclonal antibody (L2I-45) specific to the LGV L2 MOMP (48); (ii) a rabbit polyclonal anti-OmcB (kind gift from Tom Hatch, University of Tennessee); (iii) a rabbit polyclonal AsCpf1/Cas12a antibody (catalog #19984, Cell Signaling Technology); (iv) a rabbit polyclonal ant-His6 antibody (catalog #213204, Abcam); and (v) a mouse monoclonal antibody to tubulin (catalog #T5168, MilliporeSigma). The secondary antibodies used were (i) Alexa Fluor 568-conjugated goat anti-mouse IgG (catalog #A11004) from Invitrogen (Carlsbad, CA, USA) and (ii) horseradish peroxidase (HRP)-conjugated goat anti-rabbit IgG (catalog #213204, Abcam) and HRP-conjugated anti-mouse IgG (catalog A0168, MilliporeSigma).

### Cell culture and *C. trachomatis* infection

Human cervix adenocarcinoma epithelial HeLa 229 cells (ATCC CCL-2.1) were cultured in RPMI 1640 medium (Gibco) containing 5% heat-inactivated fetal bovine serum (Sigma-Aldrich), gentamicin 20 µg/mL, and L-glutamine (2 mM) (RPMI 1640–10) at 37°C in an incubator with 5% CO_2_. Cells were confirmed to be *Mycoplasma* negative by PCR as described previously (64). *C. trachomatis* strains used are listed in Table S1. The strains were authenticated by sequencing of whole PCR product of *ompA* and by staining with antibody to the LGV L2 MOMP. Spectinomycin (500 µg/mL) and cycloheximide (0.5 µg/mL) were added to propagate transformed *C. trachomatis* strains. Stocks of WT and transformed *C. trachomatis* were made every one year, and aliquots of purified EBs were stored in −80°C until use. For infection and *C. trachomatis* analysis, cells grown in 96-well plates (catalog #655090, Greiner) were inoculated with isolated EBs with a dose that results in ~30% to 40% of cells being infected, centrifuged with a Beckman Coulter model Allegra X-12R centrifuge at 1,600 × *g* for 45  min at 37°C, and cultured in RPMI 1640–10 without cycloheximide at 37°C for various times as indicated in Results. Fresh medium was added to the infected cells and incubated at 37°C for various time periods as indicated in each experimental result. For comparison, different strains were infected side-by-side in the same culture plate with a setup of at least triplicate wells per condition.

### Plasmids and transformation

Plasmids and primers used in this study are listed in Tables S1 and S2 in the supplemental material. The spectinomycin resistance encoding empty vector CRISPRi plasmid, pBOMBL12CRia(e.v.)::L2 (aka pBOMBL-As_ddCpf1vaa::L2) (22), was digested with BamHI and treated with alkaline phosphatase. Two nanograms of the *topA*-targeting or non-targeting crRNA gBlock (Table S2) was mixed with 25 ng of the BamHI-digested pBOMBL12CRia(e.v.)::L2 in a HiFi reaction (NEB) according to the manufacturer’s instructions. The reaction mix (2 µL) was then used to transform 25 µL of chemically competent 10-beta cells (C3019H, NEB), which were subsequently plated on lysogeny broth agar plates containing 50-µg/mL spectinomycin. Individual colonies were screened for the presence of the correct plasmids after miniprep (Qiagen kit) extraction from overnight cultures using restriction enzyme digest and Sanger sequencing. For the complemented vector, *topA-His_6_* was PCR amplified using DNA Phusion polymerase, the primer pair (topA/(dCas12vaa)/5′ and *topA*_6xH/(pL12CRia)/3′) (Table S2), and *C. trachomatis* serovar L2/434/Bu genomic DNA as template. The PCR product was confirmed for correct size by agarose gel electrophoresis and purified using a PCR purification kit (Qiagen). The vector pBOMBL12CRia(*topA*)::L2 was digested with SalI and treated with alkaline phosphatase as described above. The purified PCR product of *topA-His6* (13 ng) was mixed with 25 ng of the SalI-digested pBOMBL12CRia(*topA*)::L2 in a HiFi reaction. *E. coli* 10-beta transformants were obtained and plasmids were verified as described above. Two micrograms of sequencing verified CRISPRi plasmids was used to transform *C. trachomatis* serovar L2 lacking its endogenous plasmid (−pL2) as described previously (22, 26) and using 500-µg/mL spectinomycin as selection. DNA was extracted from chlamydial transformants to verify plasmid size and sequence.

### Microscopy analysis

Automated live-cell images in 96-well culture plates were acquired using an imaging reader Cytation1 (BioTek Instrument). Gen5 software was used to process and analyze the inclusion morphology (e.g., inclusion size, numbers, and mean fluorescence intensity [MFI]). For indirect IFA, the *C. trachomatis*-infected cells were fixed with 4% (wt/vol) paraformaldehyde dissolved in phosphate-buffered saline (PBS) (pH 7.4) for 15 min at room temperature, permeabilized with 0.1% (vol/vol) Triton X-100 for an additional 15 min, and blocked with 2% (wt/vol) bovine serum albumin in PBS for 30 min. Then, cells were incubated with the indicated primary antibody overnight at 4°C, followed by incubation with Alexa Fluor 488/568-conjugated secondary antibody for 45 min at 37°C. 4′,6-Diamidino-2-phenylindole was used to label DNA. In some experiments, cell images were visualized and photographed using an inverted fluorescence microscope (Zeiss Axio Observer D1) and analyzed with AxioVision software, version 4.8.

### *C. trachomatis* enumeration and end point one-step growth curve

To evaluate infectious EB progeny, IFU assays were performed in 96-well plates. Briefly, *C. trachomatis*-infected cells in culture plates were frozen at −80°C, thawed once, scraped into the medium, serially diluted, and then used to infect a fresh monolayer of HeLa cells. The infected cells were cultured in RPMI 1640–10 with 500-µg/mL spectinomycin and without cycloheximide at 37°C for 40 h. Cells were fixed, processed, and then stained with antibody against LGV L2 MOMP. Images were taken using fluorescence microscopy, and the inclusion numbers in triplicate wells were counted. The total EB numbers are presented as the number of IFUs per milliliter. In some experiments, the IFU value was normalized to the control and presented as percentage. For growth curves, the cultures were harvested at the time points 0, 12, 24, 30, and 48 h pi, and the same procedure was followed as described above to titrate IFUs.

### Anti-microbial susceptibility testing

MICs of Mox were tested in 96-well plates as described (63, 65). Purified EBs (10,000/well) were used to infect HeLa cell monolayers, followed by centrifugation with a Beckman Coulter model Allegra x-12R centrifuge at 1,600 × *g* for 45 min at 37°C. After the removal of supernatant, the infected cells were washed with PBS once and cultured in RPMI-10 lacking aTC and containing the increasing concentrations of Mox in a volume of 100 µL at 37°C in a humidified incubator with 5% CO_2_ for various time periods as indicated in each experimental result. *C. trachomatis* inclusions were immunolabeled with anti-MOMP antibody and enumerated using fluorescence microscopy. The MIC was defined as the lowest concentration of drug without visible *C. trachomatis* growth in the subculture.

### Nucleic acid analysis

For nucleic acid preparation, *C. trachomatis*-infected HeLa cells in 24-well plates were harvested at 15 and 24 h pi, respectively. Quick DNA/RNA miniprep kit (catalog # D7001, Zymo Research) was used to isolate DNAs and RNAs sequentially as instructed by the manufacturer. Residual DNA in the RNA samples was removed by treatment with 20-U RNase-free DNase I in-column for 30 min at room temperature and extensive washing. A total of 2  µg of RNA per sample was reverse transcribed into cDNA using the high-capacity cDNA reverse transcriptase kit (catalog # 4368814, Applied Biosystems). The Fast SYBR green master mix (Applied Biosystems) was used for qPCR assay in 20 µL of reaction mixture on a real-time PCR system (Bio-Rad) with the specific primer pairs listed in Table S2. Each sample was analyzed in triplicate in a 96-well plate. A negative control containing no *C. trachomatis* DNA was included. The PCR cycle conditions were as follows: 50°C for 2  min, 95°C for 5  min, 95°C for 3 s, and 60°C for 30 s. The last two steps were repeated for 40 cycles with fluorescence levels detected at the end of each cycle. Specificity of the primers was ensured with gel electrophoresis and with melting curve analysis. A standard curve was taken from purified *C. trachomatis* L2/434/Bu genomic DNA with serial dilutions for each gene-specific primer pair. The transcripts per genome copy were then calculated as the number of transcripts divided by the number of chlamydial genome DNA contents measured with the same primer pair or *tufA*.

### Immunoblotting and LC-MS/MS analysis

For immunoblotting, *C. trachomatis*-infected cells in 24-well culture plate were lysed directly in 8 M urea buffer containing 10 mM Tris-HCl (pH 8.0), 0.1% SDS, and 2.5% β-mercaptoethanol. The protein content was determined by a bicinchoninic acid protein assay kit (Thermal Fisher). The optimal amount of protein dissolved in SDS loading buffer was separated on a 10% SDS-polyacrylamide gel (PAGE) and transferred to a polyvinylidene difluoride (PVDF) membrane (Millipore). The membrane was incubated with appropriate primary antibodies, followed by incubation with the HRP-conjugated secondary antibody. For complementation of *topA* knockdown, cells seeded in a six-well plate were infected with *C. trachomatis* L2/*topA*-kdcom at a multiplicity of infection of 1 in the presence of 500-µg/mL spectinomycin and 1-µg/mL cycloheximide. At 10 h pi, cells were induced or not with 2-nM (4 ng/mL) aTC. At 24 h pi, protein lysates were harvested in 8 M urea buffer with nuclease added immediately before use. Protein concentrations were quantified using EZQ protein assay kit (Thermo Fisher) according to the manufacturer’s instructions. A total of 30 µg protein per sample was separated on a 12% SDS-PAGE gel and then transferred to a PVDF membrane. The protein of interest was probed using goat anti-MOMP and rabbit anti-His_6_ antibodies followed by donkey anti-goat 680 and donkey anti-rabbit 800 secondary antibodies (LICOR, Lincoln, NE). The blot was imaged on an Azure c600 imaging system.

For LC-MS/MS, *C. trachomatis*-infected HeLa cells were grown in the presence of aTC (at 5 ng/mL), harvested at 24 h pi, and rapidly lysed in 8 M urea 10 mM Tris-HCl (pH 7.6) buffer. Stepwise dialysis against the binding buffer (Tris-HCl [pH7.6], 20 mM imidazole, NaCl 400 mM) was performed. The clear supernatants were then used to bind to Ni-NTA beads overnight at 4°C. After washing three times with binding buffer, the bead-bound proteins were isolated on 10% SDS-PAGE. The band corresponding to the full-length TopA (~100 kDa) was cut from the gel, trypsinized, and analyzed by liquid cromatography with tandem mass spectrometry (LC-MS/MS) at the Proteomics Core Facility at Louisiana State University Health Science Center, New Orleans. The resulting data were used to search the *C. trachomatis* protein database (Taxon ID 272561). Two separate experiments were performed.

### Statistical analysis

Data for the assays include the mean ± standard derivation of at least three independent experiments. For multiples comparisons, one-way or two-way analysis of variance with 95% significance level was performed. GraphPad Prism was used for all analyses. Differences were considered statistically significant when the *P* value was <0.05.

## SUPPLEMENTAL MATERIAL

Supplemental material mbio.02584-23-s0001.pdf Table S1, Table S2, and Fig. S1-S12.

ASM does not own the copyrights to Supplemental Material that may be linked to, or accessed through, an article. The authors have granted ASM a non-exclusive, world-wide license to publish the Supplemental Material files. Please contact the corresponding author directly for reuse.
