# Supplementary material for: Targeted repression of topA by CRISPRi reveals a critical function for balanced DNA topoisomerase I activity in the Chlamydia trachomatis developmental cycle
Source: mBio. 2024 Jan 24;15(2):e02584-23. doi: 10.1128/mbio.02584-23 (PMC10865786; doi:10.1128/mbio.02584-23)
Supplement: Supplemental material — Table S1, Table S2, and Fig. S1-S12. [file mbio.02584-23-s0001.pdf]

Supplemental data

Targeted repression of *topA* by CRISPRi reveals a critical function for balanced DNA topoisomerase I activity in the *Chlamydia trachomatis* developmental cycle

Table S1 and Table S2

Figures S1-S12

Table S1. Strains and plasmids used in this study

Strains	Relevant Details	Reference
C. trachomatis		
L2/434/Bu	Bubo isolate from human with lymphogranuloma venereum (LGV).	ATCC VR-902B™
L2/*topA*-kd	Transformed LGV/L2 with a CRISPRi plasmid encoding *topA*-specific crRNA and P_{tet}-directed synthesis of dCas12 protein for *topA* knockdown	This study
L2/Nt	Transformed LGV/L2 with a plasmid encoding P_{tet}-directed synthesis of dCas12 protein and non-targeting crRNA as a vector control	This study
L2/*topA*-kdcom	Transformed LGV/L2 with a plasmid encoding P_{tet}-directed synthesis of dCas12 and TopA-His6 for complementation during *topA* knockdown	This study
L2/*topA*H6	Transformed LGV/L2 with a plasmid encoding P_{tet}-directed synthesis of *topA-his6* and lacking CRISPRi components for *topA-his6* overexpression	This study
L2/pBOMBLs	Transformed LGV/L2 with a plasmid as a vector control	This study
E. coli		
10-beta<br>(Catalog C3019H)	Host cell for cloning (*araD139D(ara-leu)7697fhuA lacX74 galK (f80 D(lacZ)M15) mcrA galU recA1 endA1 nupG rpsL (Str*^{r}*) D(mrr-hsdRMS-mcrBC)*	New England Biolabs
**Plasmid**		
pBOMBL12CRia(e.v.)::L2 (aka pBOMBL-As_dCas12::L2)	*aadA* P_{tet}-directed synthesis of dCas12 protein; P_{Nmen}::*gfp* (Spc^{r})	This study
pBOMBL12CRia(NT)::L2	*aadA* P_{Nmen}::*gfp* P_{tet}::As_dCas12vaa $P_{dnaKmut}$::As_crRNA_non-targeting (Spc^{r})	This study
pBOMBL12CRia(*topA*)::L2	*aadA* P_{Nmen}::*gfp* P_{tet}::As_dCas12vaa $P_{dnaKmut}$::As_crRNA_*topA* (Spc^{r})	This study
pBOMBL12CRia-*topA*_6xH(*topA*)::L2	*aadA* P_{Nmen}::*gfp* P_{tet}::As_dCas12vaa-*topA*_6xH $P_{dnaKmut}$::As_crRNA_*topA* (Spc^{r})	This study
pBOMBL-spc	*aadA* P_{Nmen}*::gfp* P_{tet}*::mCherry* (Spc^{r})	This study
pBOMBLs-*topAhis6*	pBOMBL-spc derived expression vector. *aadA* P_{tet}-directed synthesis of *topA-his6*; P_{Nmen}::*gfp* (Spc^{r})	This study

Table S2. Primers and gBlocks used in this study

Primer name	sequence 5'→3'	Use/features
euo-rtF	TCAAGGAGAGCTTCTGTTTGATAAC	RT-qPCR for *euo*
euo-rtR	TGCGTGTAGCATAGTAAATCTTCTG	
tuf-rtF	GTAACTCTGCCTGAGGGAATTGA	RT-qPCR for *tufA*
tuf-rtR	CACGAATCGCAAATCTCATACCT	
*gfp*_rtF	GTATACATCATGGCAGACAAACAA	RT-qPCR for *gfp*
*gfp*_rtR	TGTTGATAATGGTCTGCTAGTTGAA	
*incD*_rtF	CTCTGTAGCCCTGTTTCTGTTTTAG	RT-qPCR for *incD*
*incD*_rtR	CTAGTCACAGCTTCTGTAGTCAGCA	
*omcB*_rtF	GTTTGCGTTGCCCAGTAGTT	RT-qPCR for *omcB*
*omcB*_rtR	CACGCTGTCCAGAAGAATGA	
*hctB*_RT_F	AAACATACTGCAGCTTGTGGAC	RT-qPCR for *hctB*
*hctB*_RT_R	GAGCTGTACGAGAACGGTTAGG	
rt*ct190/189*PrF	GAGTCACGCTTTATCCATTCGG	RT-qPCR for *gyrB/gyrA*
rt*ct190/189*PrR	AGCTCTCCTTCATTTCCTCTTCA	
rt*ct643*prF	GTTGAATCCCCAGCCAAGATTA	RT-qPCR for *topA* and sequencing
rt*ct643*prR	CCCTTTTGCAGGAAGATCAACA	
rt*ct660/661*PrF	GAGAATCTTGTTACCAACCTCTAGC	RT-qPCR for *pare/parC*
rt*ct660/661*PrR	GTTCCAAAATGACGTAAGACGC	
*topA*_rtF2	CAATCGCATACCAAGCCTTT	RT-qPCR for *topA* and sequencing
topA rtR2	ACGTAGCCTTTGCCCTTCTTT C	
topA/(dCas12vaa)/5'	cgcaacgtagctgcttaagtaccgaggagaatctgcATGAAAAAATCCTTAATCATTG	Cloning *topA-his6* into SalI site of pL12CRia (*topA*)
*topA*_6xH/(pL12CRia)/3'	catgagcggatacatatttgaatggttaatggtgatggtgatggtgCTCTTCCTTGATTAAGTGCG	
topA/(pBOMBL)/5'	aaagatcttcacacaggacatctgcATGAAAAAATCCTTAATCATTG	Cloning *topA-his6* into EagI/KpnI sites of pBOMBLs (used with *topA*_6xH/(pL12CRia)/3'

gBlock Name	sequence 5’→3’	Use/Features
topA crRNA	tgtgaaagtgggtcttaagacgtcggtactgcatgtgacgcacgtagatcatgca*TTCACCGGTGGAGACGGTTTTCTTATAATGACACC*TAATTTCTACTCTTGTAGAT**TGCGAGAGACTAAGATCCCGC**CAAATAAAACGAAAGGCTCAGTCGAAAGACTGGGCCTTTCGTTTTATcaacagcggtctactgaatctgagctagtgcgtgatataattaaaattatattca	For CRISPRi knockdown of *topA;*<br><br>Lower case for plasmid overlap and spacer, *italicized* for P_{dnaK} sequence, underlined for crRNA scaffold, **bold** for *topA* targeting sequence, Upper case for *rrnB*1 terminator
Non-targeting crRNA	tgtgaaagtgggtcttaagacgtcggtactgcatgtgacgcacgtagatcatgca*TTCACCGGTGGAGACGGTTTTCTTATAATGACACC*TAATTTCTACTCTTGTAGAT**ACCGAGTTGCCCGTTAAAGTA**CAAATAAAACGAAAGGCTCAGTCGAAAGACTGGGCCTTTCGTTTTATcaacagcggtctactgaatctgagctagtgcgtgatataattaaaattatattca	Non-targeting CRISPRi control;<br><br>Lower case for plasmid overlap and spacer, *italicized* for P_{dnaK} sequence, underlined for crRNA scaffold, **bold** for *non-targeting* targeting sequence, Upper case for *rrnB*1 terminator

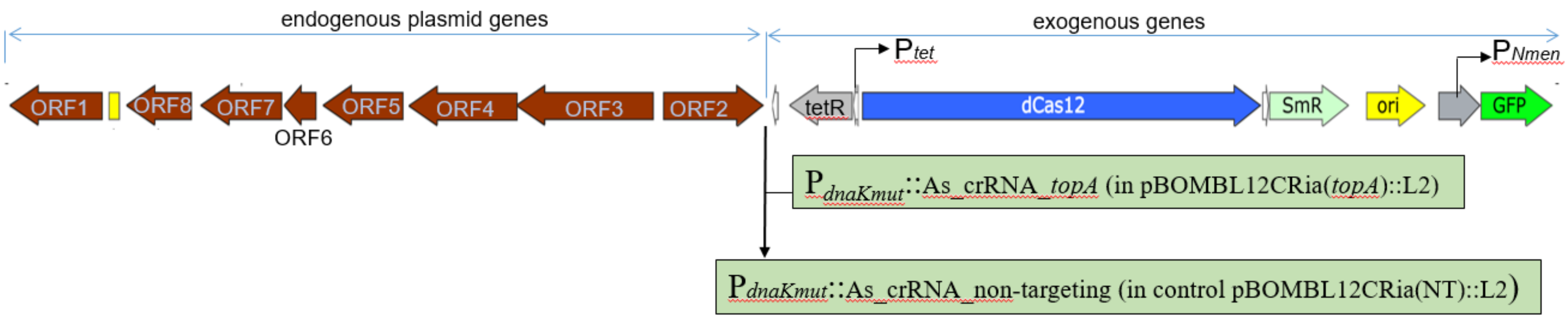

Figure S1. Depiction of the general construct of the pBOMBL12CRia-derived shuttle plasmids that were used for *C. trachomatis* transformation. The coding sequence of the *dCas12* gene is placed downstream of a *tet* repressor-regulated promoter in an expression vector and an ATG triplet serves as the initiation codon. Whereas pBOMBL12CRia(*topA*)::L2 contains a chlamydial *topA*-specific crRNA, the pBOMBL12CRia(NT)::L2 does not.

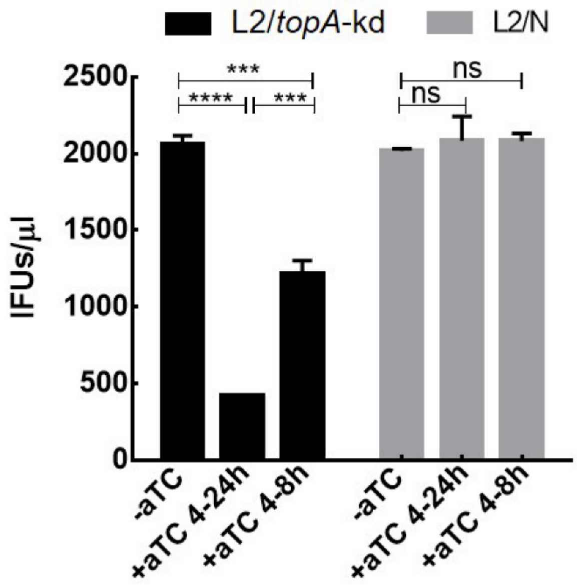

Figure S2. Numeration of EBs using IFU assay. *C. trachomatis* L2/*topA*-kd or L2/Nt infected HeLa cells were cultured for 20 or 4 h in the medium containing aTC starting at 4 h pi, harvested, and subjected to IFU assay. Triplicate results in a representative experiment are shown as mean ± SD. At least four independent experiments were performed. Statistical significance was determined by two-way ANOVA (simple effects within columns) followed by Tukey's post-hoc test. ****$P \leq 0.0001$; ***$P \leq 0.001$, ns: no significance. (Also see Fig. 2d for relative IFUs).

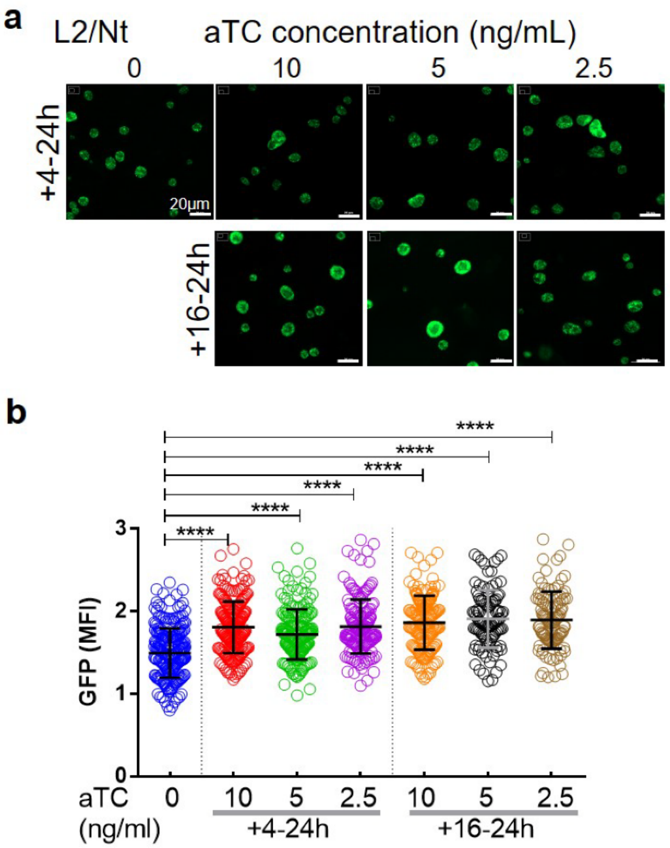

Figure S3. (**a**) Live-cell images of *C. trachomatis* L2/Nt that has a wild type *topA* lacking the *topA*-specific crRNA. HeLa cells were infected with *C. trachomatis* L2/Nt at multiplicity of infection ~0.4 and cultured in aTC free medium. The increasing concentrations of aTC (0, 2.5, 5, or 10 ng/mL) were added starting at 4 h (upper panels) or at 16 h pi (lower panels). The automated imaging was taken at 24 h pi under the same exposure conditions using Cytation 1. Scale bar = 20 µm. (**b**) Quantifying GFP MFI of the single chlamydial inclusions. Values of GFP MFI are presented as mean ± SD from the individual inclusion numbers equal to 166 ± 50 per condition in replicate wells. ****$P \leq 0.0001$, comparison was made using one-way ANOVA followed by Tukey's post-hoc test. Note: varied, but no decrease in GFP was evident in the presence of aTC.

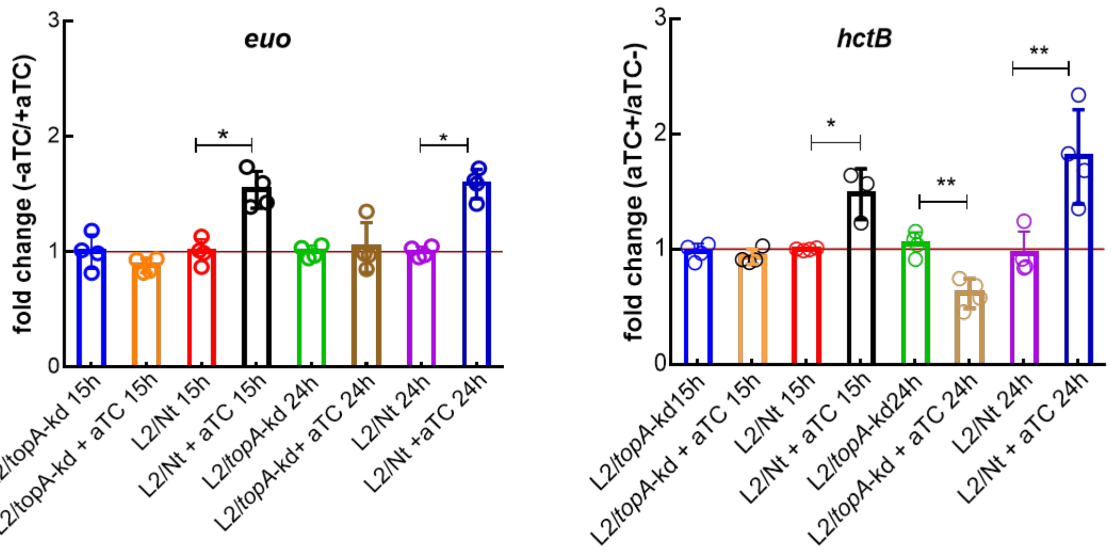

Figure S4. Quantifying transcript levels of *euo* and *hctB* in *C. trachomatis* strains, L2/*topA*-kd or L2/Nt (control) using RT-qPCR. The mRNA transcript levels were normalized to the gDNA control as determined by qPCR targeting chlamydial *euo* (left) or *hctB* (right). Values were presented as mean ± SD of four biological replicates. The data are presented as the ratio of transcript in the presence of aTC to that in the absence of aTC, which is set at 1 as shown by a red line. Statistical significance was determined by one-way ANOVA followed by Tukey's post-hoc test. *$P \leq 0.05$, **$P \leq 0.01$.

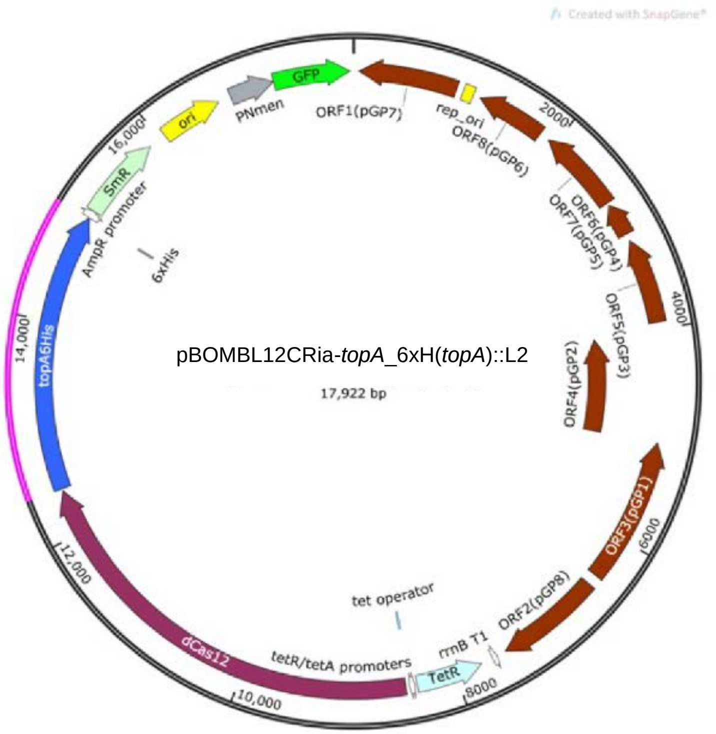

Figure S5. Map of expression vector pBOMBL12CRia-*topA*_6xH(*topA*)::L2 showing the relevant vector elements, including P_{tet}-controlled dCas12 and *topA*-*his6*, as indicated. This plasmid was transformed into *C. trachomatis* using spectinomycin as a selection agent resulting in strain L2/*topA*-kdcom that was used for complementation.

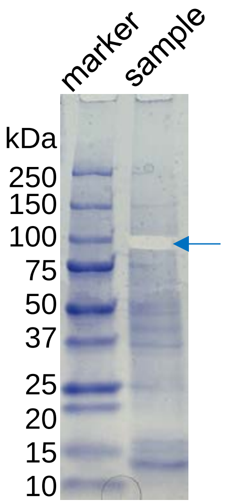

>TopA
MKKSLIIVESPAKIKTLRKLLGEGFIFDSSLGHIVDLPAKGFGIDIENGFVPDYQIL
EGKEEVIRKICAEAKKCDVVYLAPDPDREGEAIAWHIANQLPKDTKIQRISFNA
ITKGAVTEALKHPREIDMALVNAQQARRFLDRIVGYKISPILGRKLQRWSGVS
AGRVQSVALKLVVDREYAIERFVPVEFWNIRVHLKDPQTQKTFWAHLHSVN
GKKWEKEIPEGKTSDEVILIDSKEKADEIVALLESATYVVDRVESKEKKRHAYPP
FITSTLQQEASRHYRFSSSRTMNIAQTLYEGVDLDSQGAVGLITYMRTDSVRT
DPEAVKQVRKYIEGHFGKEFVPSSPNVYATKKMAQDAHEAIRPTDVTITPESI
RSKLTEDQYKLYSLIWKRFVASQMISAIYDTLAIRITTNKGIDLRATGSCLKFKG
FLAVYEEKRDEEGDEEENIHLPKLNERDVLTKEELEAEQSHTKPLPRFTEASLV
KELEKSGIGRPSTYATIMNKIQSREYTLKEGQRLRPTELGKVVCQFLETNFPRI
MDIGFTAGMEDELELIADNKKPWKQLLQEFCELFLPFVVTAEKEAFIPRIVTEI
DCPKCHKGKLVKIWAKNRYFFGCSEYPTCDYKTSEEELTFDKNEYAEDTPWD
APCALCGGEMKVRHGKFGSFLGCENYPKCHYIVNLFKKGEAGAEPEATVHCP
AEGCTGHLVKRRSRFNKMFYSCSEYPACSVIGNSVDAVIEKYAGTPKTPYEKK
PKAKKSIASTKGKAAKTVKKSSATTKKRATKAYTPSAALAAVIGADPVGRPEA
TKKLWEYIKEKGLQSPQNKKIIIPDSKLQGVIGADPIDMFALSKKLSAHLIKEE

Peptides found in experiment 1
[R].EIDMALVNAQQAR.[R]
[R].FVPVEFWNIR.[V]
[R].FVASQMISAIYDTLAIR.[I]

Peptides found in experiment 2
[R].EIDMALVNAQQAR.[R]
[K].TSDEVILIDSK.[E]
[R].FVASQMISAIYDTLAIR.[I]

Figure S6. Analysis of TopA-His6 by sodium dodecyl-sulfate polyacrylamide gel electrophoresis (SDS-PAGE) and liquid chromatography with tandem mass spectrometry (LC-MS/MS). *C. trachomatis* strain, L2/*topA*-kdcom or L2/Nt, infected HeLa cells grown in the presence of aTC (at 5 ng/mL) were harvested at 24 h pi and rapidly lysed in 8M urea 10mM Tris-HCl (pH 7.6) buffer. After step-wise dialysis into the binding buffer (Tris-HCl (pH 7.6), 20mM imidazole, NaCl 400mM), the clear supernatants were used to bind to Ni-NTA beads overnight at 4 ˚C. Following washing three times with binding buffer, the proteins were separated on SDS-PAGE gel. The enrichment of a band corresponding to 100kDa was observed in L2/*topA*-kdcom, but not in L2/Nt (not shown). This band was cut and subjected to LC-MS/MS. The data were used to search for *C. trachomatis* protein database (Taxon ID 272561). The peptides covering ~8% of TopA amino acids were identified in two separate experiments. These data verify the inducible expression of TopA-His6 in *C. trachomatis.*

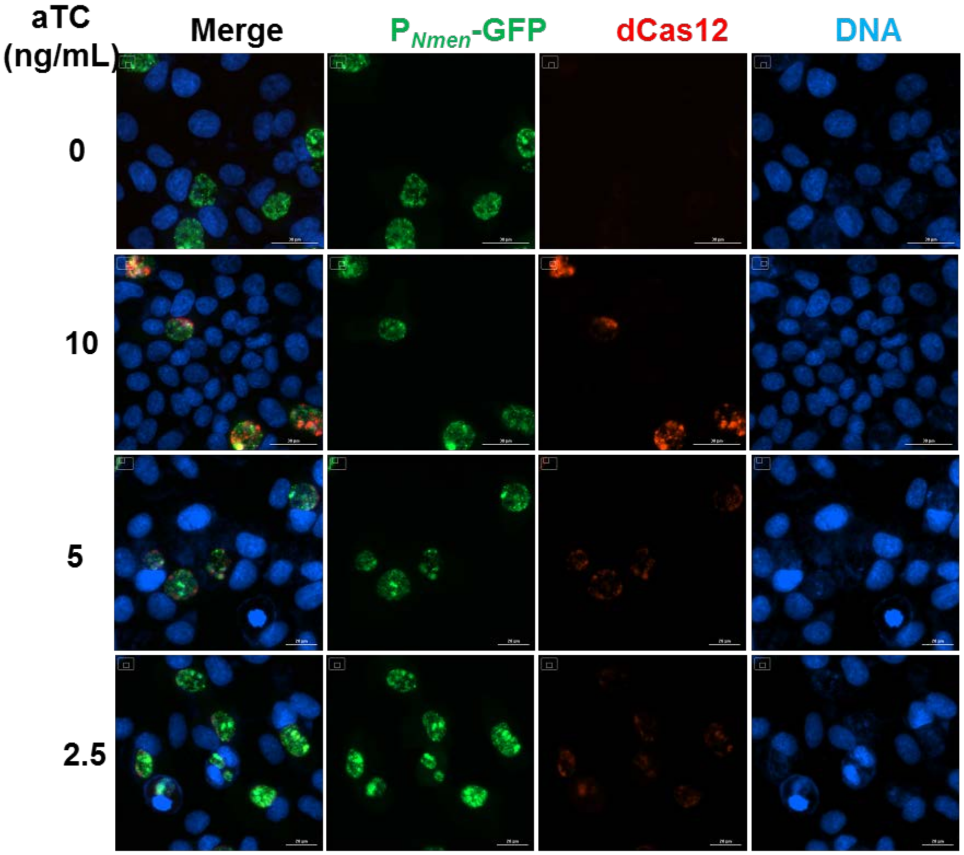

Figure S7. Immunofluorescence micrographs of *C. trachomatis* L2/*topA*-kdcom expressing dCas12. Cells were grown in the presence of increasing concentrations of aTC (0, 2.5, 5, and 10 ng/mL) and fixed at 40 h pi for IFA. The dCas12 was immunolabelled with rabbit anti-dCas12 antibody and visualized with Alexa Fluor 568-conjugated goat anti-rabbit IgG. DAPI-stained DNA (blue) and *C. trachomatis* expressing GFP (green) and dCas12 (red) are shown. Scale bar=30µm.

Figure S8

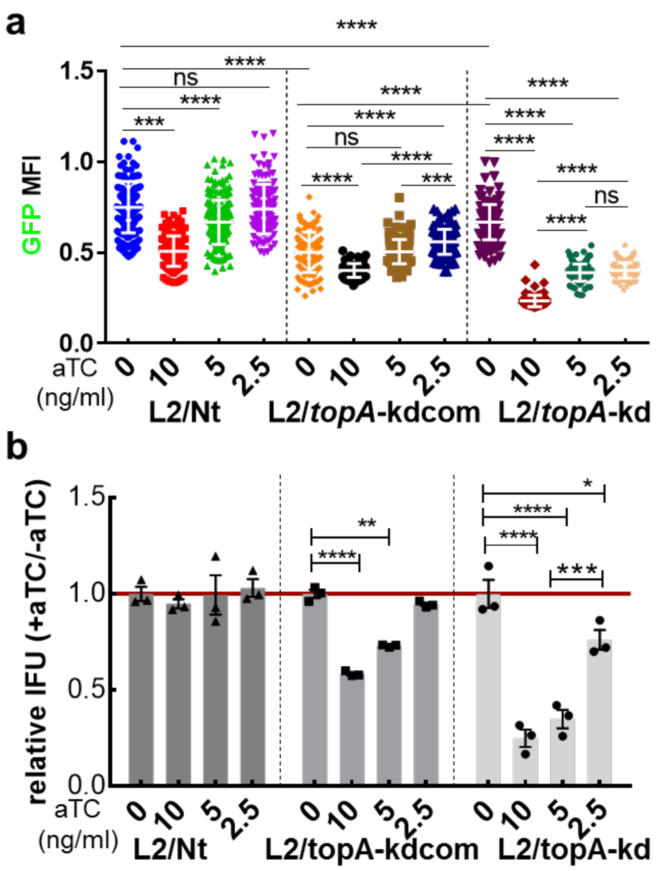

Figure S8. Determination of the effect of aTC addition on the growth of *C. trachomatis* L2/*topA*-kdcom. HeLa cells infected with L2/*topA*-kdcom and the control strains, L2/*topA*-kd and L2/Nt, were cultured in the RPMI-10 containing the increasing amounts of aTC (at 0, 2.5, 5, and 10 ng/mL) starting at 4 h pi. **(a)** Analysis of the levels of P_{Nmen}-GFP MFI. Live-cell imaging in combination with green fluorescence and bright light detection were obtained at 24 h pi using Cytation1. Individual chlamydial inclusions equal to 142.5 ± 35 per condition were measured using Gen 5 software. The GFP MFI is presented as the intensity ratio of GFP to bright light field in the same individual inclusion. (**b**) Numeration of EBs. *C. trachomatis* infected cells cultured for 40 h were used for IFUs assay. Values are presented as the ratio of IFUs from aTC-exposed sample to that from aTC-unexposed sample, which is set at 1 as shown by a red line. Triplicate results in a representative experiment are shown as mean ± SD. At least four independent experiments were performed. Statistical significance was determined by one-way ANOVA followed by Tukey's post-hoc test. *$P \leq 0.05$, ** $P \leq 0.01$, ***$P \leq 0.001$, ****$P \leq 0.0001$, ns: no significance.

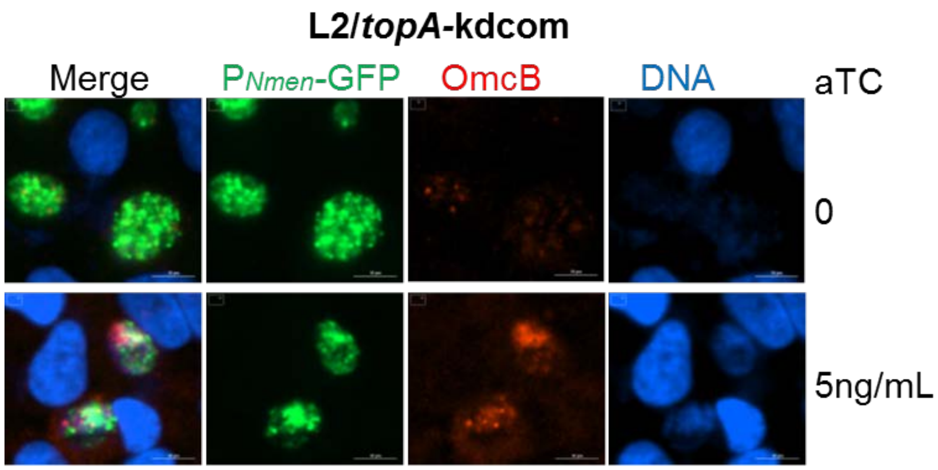

Figure S9 Immunofluorescence micrographs of *C. trachomatis* L2/*topA*-kdcom expressing OmcB. Infected cells were grown in the absence or presence of aTC (at 5 ng/mL) and fixed at 24 h pi for IFA. The OmcB protein was immunolabelled with rabbit anti-OmcB antibody and visualized with Alexa Fluor 568-conjugated goat anti-rabbit IgG. DAPI-stained DNA (blue) and *C. trachomatis* expressing GFP (green) and OmcB (red) are shown. Scale bar = 10μm. Note: the enhancement of the inclusion-associated OmcB signal induced by aTC addition compared to that of mock aTC addition.

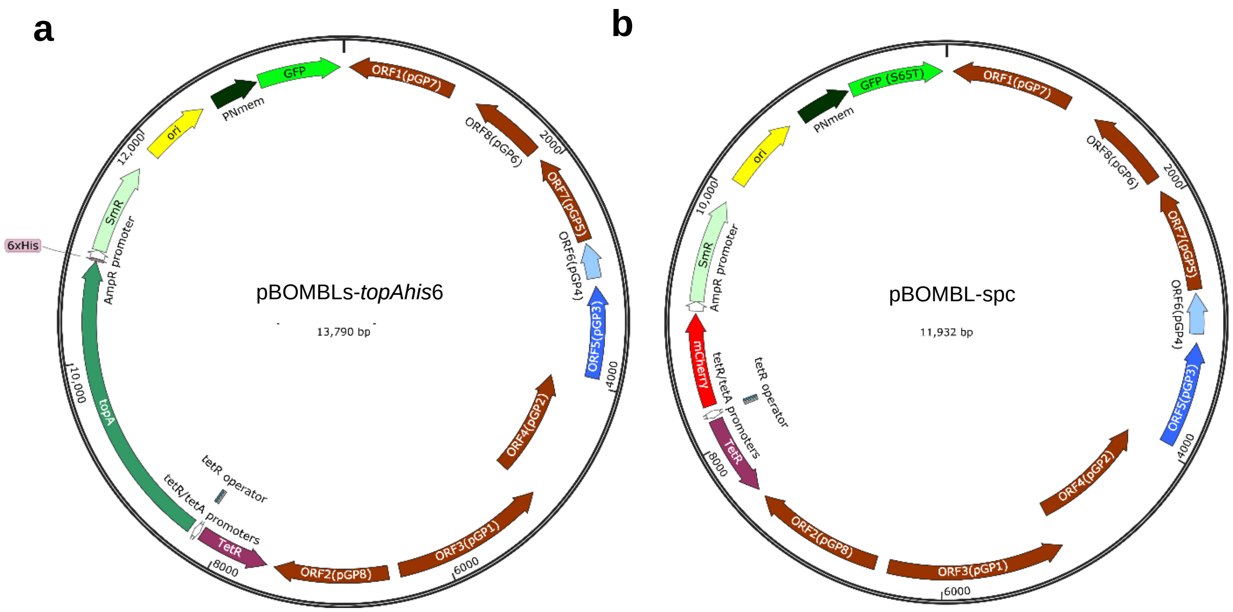

Figure S10. **(a)** Map of expression vector pBOMBLs-*topAhis6* showing the relevant elements, including P*tet*-controlled *topA*-*his6* and P*Nmem*-GFP. This plasmid was transformed into *C. trachomatis* resulting in strain L2/*topA*H6 that was used for *topA-his6* overexpression study. (**b**) Map of control plasmid pBOMBL-spc. This plasmid was transformed into *C. trachomatis* resulting in strain L2/pBOMBLs that was used in control experiment.

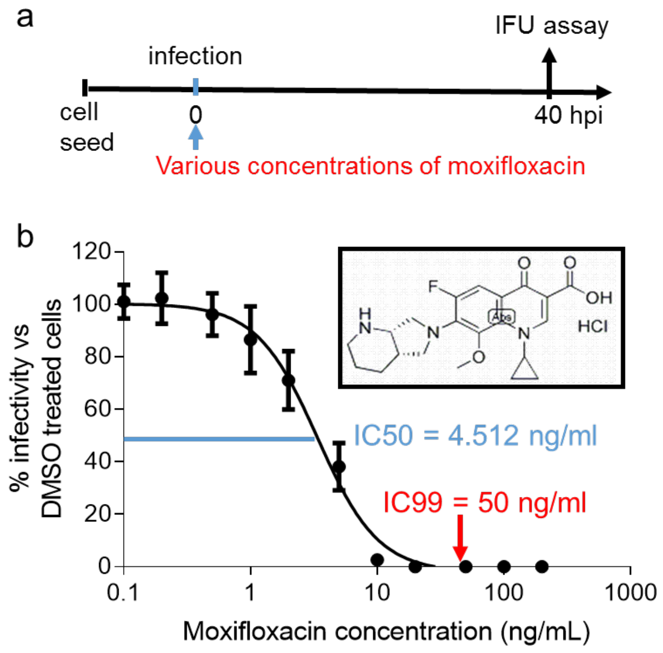

Figure S11. Determining minimal inhibitory concentration (MIC) of moxifloxacin against *C. trachomatis*. (**a**) Schematic diagram of the experimental procedure. Moxifloxacin was added into the *C. trachomatis* culture immediately after infection (0 h pi). (**b**) MIC of moxifloxacin. Structure of moxifloxacin is shown. *C. trachomatis* infected HeLa cells were cultured in medium containing increasing concentrations of moxifloxacin (at 0.1, 0.25, 0.5, 1, 2.5, 10, 25, 50, 100, and 400 ng/mL) for 40 h prior to the analysis of IFUs by passaging on a fresh monolayer of HeLa cells in the absence of antibiotic. The relative infectivity (y-axis) normalized to DMSO control was presented as percentage (mean ± SD). The moxifloxacin concentrations used are shown on the x-axis. Studies were repeated three times in quadruplicate.

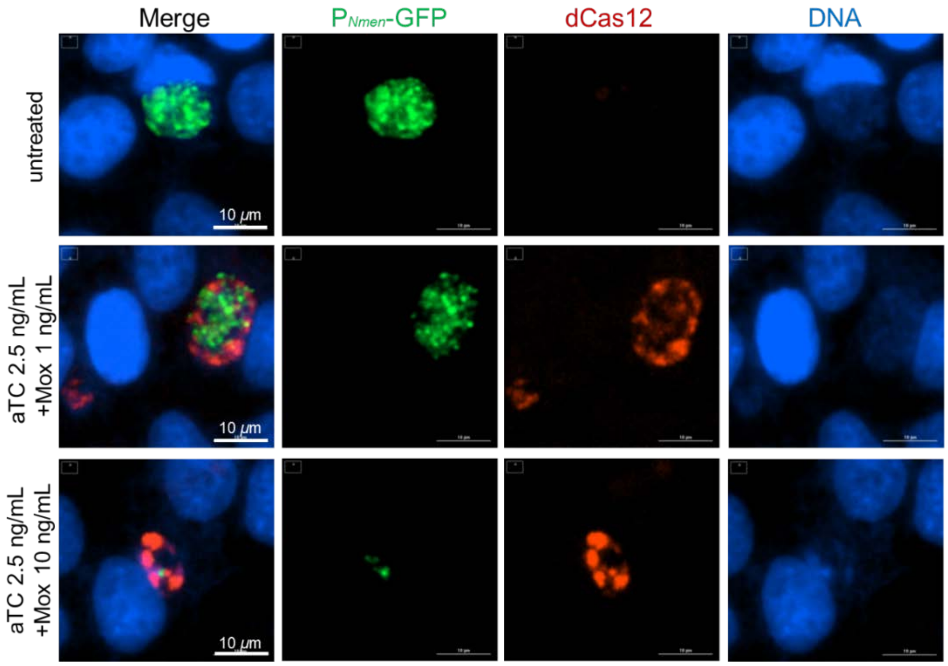

Figure S12. Immunofluorescence micrograph of *C. trachomatis* L2/*topA*-kdcom expressing dCas12. HeLa cells with infection of *C. trachomatis* were cultured in the presence of aTC (at 2.5 ng/mL) and Mox (at 1 ng/mL or 10 ng/mL) for 40 h pi and fixed for IFA. The dCas12 was immunolabelled with rabbit anti-dCas12 antibody and visualized with Alexa Fluor 568-conjugated goat anti-rabbit IgG. DAPI-stained DNA (blue) and *C. trachomatis* expressing GFP (green) and dCas12 (red) are shown. Scale bar=10μm.
